# Effect of percutaneous coronary intervention on chronic total occlusions with documented viability or ischemia: a systematic review and meta-analysis

**DOI:** 10.1186/s12872-025-05405-0

**Published:** 2025-12-04

**Authors:** Luís Leite, Tomás Carlos, Gonçalo Ferraz Costa, Inês Cruz, Helena Donato, Rodolfo Silva, Miguel Castelo-Branco, Lino Gonçalves, Maria João Ferreira

**Affiliations:** 1 Cardiology Department, Coimbra Hospital and University Centre, Unidade Local de Saúde de Coimbra, Coimbra, Portugal; 2 Institute for Nuclear Sciences Applied to Health (ICNAS), University of Coimbra, Coimbra, Portugal; 3 Faculty of Medicine, University of Coimbra, Coimbra, Portugal; 4 Coimbra Institute for Biomedical Imaging and Translational Research (CIBIT), Coimbra, Portugal; 5 Documentation and Scientific Information Service, Coimbra Hospital and University Centre, Unidade Local de Saúde de Coimbra, Coimbra, Portugal; 6 Coimbra Institute for Clinical and Biomedical Research (iCBR), Coimbra, Portugal

**Keywords:** Chronic total coronary occlusions, Percutaneous coronary intervention, Viability, Ischemia, Cardiac magnetic resonance, Positron emission tomography

## Abstract

**Background:**

Percutaneous coronary intervention (PCI) for chronic total occlusions (CTO) is common despite the equivocal evidence regarding its benefits. This study aimed to evaluate the impact of pre-PCI viability or ischemia assessment on left ventricular (LV) function, ischemic burden, symptoms, and major adverse cardiovascular events in CTO patients.

**Methods:**

A systematic search of PubMed/MEDLINE, EMBASE, CENTRAL, Web of Science Core Collection, ClinicalTrials.eu, and ClinicalTrials.gov was conducted. Studies assessing viability and/or ischemia before PCI with follow-up data were included. Quality was assessed using Cochrane Risk of Bias 2.0 and ROBINS-I tools. Meta-analyses were conducted for quantitative outcomes and narrative synthesis for heterogeneous data. A total of 21 studies (3 randomized, 18 observational) were included; notably, among the randomized trials, only one required the presence of viability or ischemia as an inclusion criterion.

**Results:**

Twenty-one studies were included in this review. Cardiac magnetic resonance was the most used imaging modality, followed by positron emission tomography. Successful PCI was associated with improved LV ejection fraction (MD: 3.97%; 95% CI: 1.51% to 6.42%) but no significant change in LV volumes. Regional segmental wall thickness increased in dysfunctional viable segments (MD: 16.70%; 95% CI: 11.15% to 22.26%), but not in non-viable segments. Successful CTO-PCI improved hyperaemic myocardial blood flow (MBF) (MD: 1.03 mL/min/g; 95% CI: 0.94 mL/min/g to 1.13 mL/min/g), rest MBF (MD: 0.10 mL/min/g; 95% CI: 0.06 mL/min/g to 0.14 mL/min/g), and coronary flow reserve (MD: 1.16; 95% CI: 1.03 to 1.30). The extent of ischemia reduction was associated with improved long-term prognosis and symptom relief.

**Conclusions:**

Pre-PCI viability and ischemia assessment may help identify patients more likely to achieve better functional recovery and symptom relief after successful CTO recanalization. These findings support its role in patient selection and highlight the need for further randomized studies to confirm prognostic value.

**Trial registration:**

The review protocol was registered in PROSPERO (ID: CRD42023426858).

**Supplementary Information:**

The online version contains supplementary material available at 10.1186/s12872-025-05405-0.

## Background

Chronic total coronary occlusions (CTO) are present in 16–20% of patients with coronary artery disease undergoing coronary angiography [1, 2] and represent one of the most challenging scenarios in percutaneous coronary intervention (PCI). Although technical success rates have improved substantially due to advances in devices and operator expertise, the clinical benefit of CTO-PCI remains debated [3].

Current guidelines for managing CTO revascularization reflect ongoing uncertainty in the supporting evidence. The 2021 ACC/AHA/SCAI guidelines [4] downgraded CTO-PCI to a Class IIb recommendation due to randomized clinical trials (RCTs) that have not demonstrated improved function and have been equivocal regarding symptoms. In contrast, the 2024 European clinical consensus statement (EAPCI/EACVI/ESC Working Group on Cardiovascular Surgery) [5] supports CTO-PCI in patients with angina or dyspnea resistant to optimal medical therapy (OMT) or with significant ischemia/viability in the CTO territory, particularly when LV dysfunction is present. While these recommendations emphasize the importance of ischemia and viability documentation before proceeding to PCI, many studies assessing the benefits of CTO-PCI often lack data on ischemia burden and viability.

To date, no systematic review has comprehensively examined whether viability- or ischemia-guided PCI for CTOs influences functional recovery, symptom relief, or clinical outcomes. This knowledge gap is critical for patient selection and personalized treatment strategies. This review aimed to determine whether, in patients with chronic total occlusions (Population), PCI (Intervention), compared with either OMT, unsuccessful PCI, or baseline status (Comparators), improves LV function, ischemic burden, symptoms, and major adverse cardiovascular events (MACE) (Outcomes), over short- to mid-term follow-up (Timeframe), based on randomized and observational studies (Setting).

## Methods

This systematic review and meta-analysis were conducted in accordance with the Preferred Reporting Items for Systematic Reviews and Meta-Analyses (PRISMA) guidelines [6] (Supplementary Table 1). The study protocol was registered with PROSPERO (CRD42023426858) and published before study commencement [7].

### Eligibility criteria

We aimed to identify RCTs, cohort studies (prospective or retrospective), cross-sectional studies, and case-control studies in which patients with established CTO underwent viability and/or ischemia testing before the decision to perform PCI, followed by post intervention testing or clinical endpoint follow-up. The diverse testing modalities encompassed cardiac magnetic resonance (CMR), positron emission tomography (PET), stress echocardiography, or single-photon emission computed tomography (SPECT). Studies lacking viability and/or ischemia testing before PCI or lacking clinical endpoints or postintervention testing were excluded. Supplementary Table 2 presents our PICO description. For studies with multiple publications, we selected the one with the largest sample size or the most suitable data for our objectives. There were no restrictions on publication year, status, or language.

### Information sources

A comprehensive search was conducted across the following databases: PubMed/MEDLINE, EMBASE, Cochrane Central Register of Controlled Trials (CENTRAL), and Web of Science Core Collection. We also searched ClinicalTrials.gov and ClinicalTrials.eu for ongoing or unpublished trials. Gray literature was also queried to include all relevant studies. Additionally, we manually searched reference lists of included studies and previously published systematic reviews on CTO-PCI.

### Search strategy

A medical librarian with expertise in systematic reviews developed the search strategy. It incorporated controlled vocabulary (e.g., Medical Subject Headings [MeSH] terms) and text word searches tailored to each database. Boolean operators (“AND” and “OR”) were used to merge the search terms effectively. The detailed search strategy for each database is provided in Supplementary Tables 3a to 3d.

### Selection process

Studies and literature reviewed were imported to Endnote Reference Library (Version 21; Clarivate Analytics, Philadelphia, PA, USA), where duplicates were removed. Two reviewers (L.L. and T.C.) independently screened studies by title and abstract, then reviewed full-text articles based on predefined inclusion and exclusion criteria, with disagreements resolved by a third reviewer (M.J.F.). Inter-rater agreement for study selection was strong (Cohen’s kappa = 0.89).

### Data collection process

A standardized data extraction form was developed and piloted. Two reviewers (L.L. and T.C.) extracted the following data: study identification, sample size, study design, number of eligible patients, demographic information, type of cardiac exam conducted pre- and post-intervention, definition of viability or ischemia assessment for each study, intervention type (only successful PCI, PCI vs. no PCI, successful PCI vs. unsuccessful PCI), outcome measured, and main results. Disagreements were resolved by consensus or with a third reviewer (M.J.F.).

### Data items

Eligible outcomes were categorised as follows:

- LV function: Regional systolic wall thickening (SWT) in dysfunctional segments supplied by the CTO vessel, LV ejection fraction (LVEF), LV end-diastolic volume (LVEDV), and LV end-systolic volume (LVESV).
- Ischemic burden: Myocardial blood flow (hyperaemic and rest), coronary flow reserve, and perfusion defect size.
- Symptoms/Quality of life: Six-Minute Walk Test (6MWT), Seattle Angina Questionnaire (SAQ), Questionnaire Short Form 36 Health Survey (SF-36), and New York Heart Association (NYHA) class.
- Major adverse cardiac events (MACE): All-cause mortality, nonfatal myocardial infarction (MI), and clinically driven repeat revascularization.

Although some variations existed between the follow-up duration, outcomes were assessed at baseline and post-intervention. We also extracted whether ischemia and/or viability testing was used as a prospective eligibility criterion for PCI or was assessed retrospectively for analysis purposes.

### Risk of bias assessment

The risk of bias in individual studies was assessed by two reviewers (L.L. and T.C.), with disagreements resolved by a third reviewer (G.C.). We evaluated the risk of bias in included studies using two established tools, accommodating the diverse study designs. RCTs were assessed using the Cochrane Risk of Bias 2.0 tool, which examines biases across several domains including randomization, allocation concealment, blinding of participants and personnel, blinding of outcome assessment, incomplete outcome data, selective reporting, and other biases. Non-randomized studies were evaluated with the ROBINS-I tool, designed to assess bias in estimates of the effects of interventions in terms of confounding, participant selection, classification of interventions, deviations from intended interventions, missing data, measurement of outcomes, and reported results. Each study’s bias risk was independently reviewed and categorized by each reviewer, with discrepancies resolved through consensus. Inter-rater agreement for risk-of-bias assessment was substantial (Cohen’s kappa = 0.83).

### Synthesis methods

Our synthesis approach varied depending on the data available. For outcomes amenable to quantitative synthesis, we conducted meta-analyses using appropriate statistical models, which are detailed further in the statistical analysis section. Studies were included in the meta-analysis only if they reported outcomes with sufficient statistical detail (e.g., mean ± SD, sample size, and measures of variability) and were comparable across at least two studies. Studies reporting unique endpoints or presenting non-numerical or incomplete data were synthesized narratively. This approach organized findings by outcome measures, type of intervention, and the presence of viability and/or ischemia testing, highlighting trends and noting substantial differences. The certainty of evidence for each outcome was evaluated using the Grading Recommendations Assessment, Development and Evaluation (GRADE) system, which considers study limitations, consistency of effect, imprecision, indirectness, and publication bias.

### Statistical analysis

Statistical analyses were performed using Review Manager, Version 5.4.1 (Cochrane Collaboration, Oxford, UK). For continuous data, mean differences (MD) with 95% confidence intervals (CIs) were calculated using a random effects generic inverse variance method. Heterogeneity among studies was assessed using Higgins I^2^ statistic, with 25%−50% values indicating mild heterogeneity, 50%−75% moderate, and over 75% high heterogeneity. A p-value less than 0.05 was considered statistically significant for all tests. The random effects model was chosen due to the expected variation across studies regarding populations, interventions, and outcomes measured. Sensitivity analyses were also conducted to explore the potential influence of study design and inclusion criteria on heterogeneity, and to assess the robustness of the pooled estimates.

### Reporting bias assessment

To assess the presence of publication bias, funnel plots (Supplementary Fig. 1 - Supplementary Fig. 5) were constructed for each meta-analysis outcome and examined for asymmetry. An asymmetrical appearance would suggest potential underreporting of smaller or non-significant studies.

### Ethics and dissemination

This systematic review utilized publicly available, published data, so no ethical approval was required.

## Results

### Study selection

**Fig. 1:**
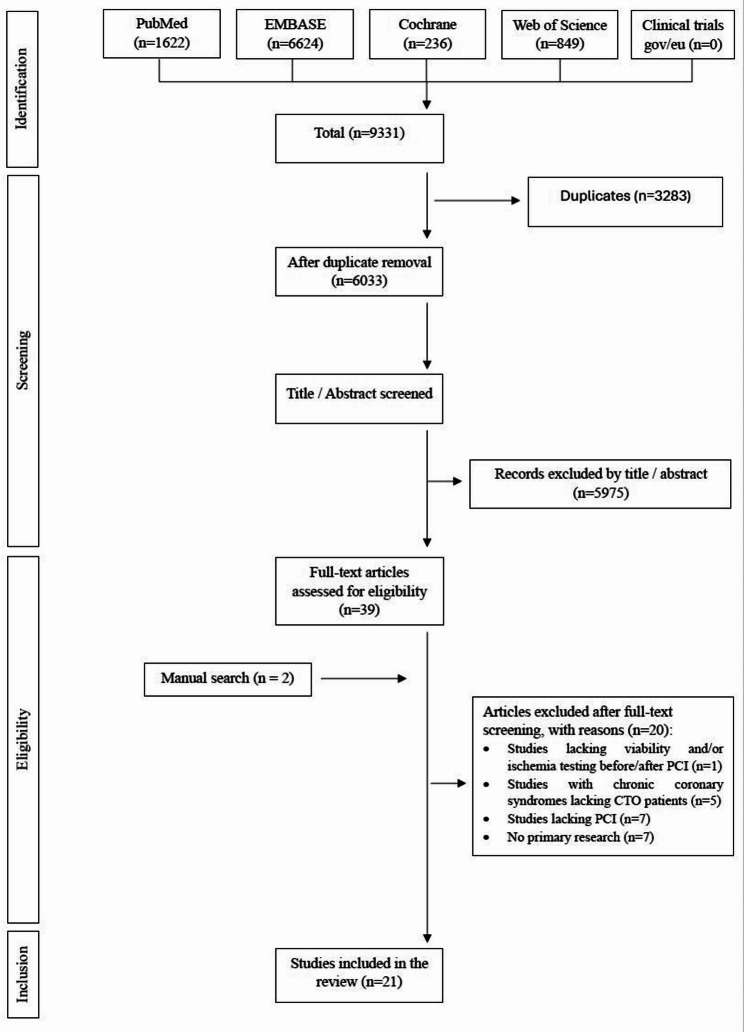
Flow Diagram of Study Selection. CTO: Chronic total coronary occlusion; PCI: Percutaneous coronary intervention

A comprehensive search of the databases yielded 9331 records. After removing duplicates, 6033 records were screened, and 39 full-text documents were reviewed. We then expanded our search to include studies cited by any of the initially included papers, as well as the references of these studies. This led to the inclusion of two additional articles, bringing the final total to 21 studies. A flow diagram of the study selection process is shown in Fig. 1.

Of the 21 included studies, 11 provided quantitative data suitable for meta-analysis. Studies were included in meta-analysis only if they reported outcomes with sufficient statistical detail (e.g., mean ± SD, sample size, and measures of variability) and were comparable across at least two studies. The remaining 10 studies either reported outcomes in formats not amenable to pooling or addressed outcomes that were unique and not reported by other studies. These studies were included in the qualitative (narrative) synthesis only.

### Study characteristics

The characteristics of the included studies are summarized in Table 1. Of the 21 studies, three were RCTs comparing CTO-PCI to OMT. Eighteen were observational studies, most of which included only patients with successful CTO-PCI, with two comparing successful to unsuccessful CTO-PCI. The study of Obedinskiy et al. [8] focused exclusively on patients with right coronary artery (RCA) CTO, while the remaining studies included CTO in any coronary artery.

**Table 1:** Study design features and key characteristics

First author, year	Study design	Sample size (male %)	Age (mean ± SD, years)	CTO artery	Type of evaluation	Ischemia or viability evidence required	Functional imaging test	Intervention	Outcome (1 - LV function; 2 - Ischemic burden, 3 - Symptoms/Quality of life; 4 - MACE)	Follow-up (months)	Included in Meta-Analysis
Baks 2006[12]	Cohort prospective, single-center	27 (81.5%)	64 ± 10	LAD 14, RCA 10, Cx 3	Viability	No	CMR	Successful PCI	1	5	Yes
Bucciarelli-Ducci 2016[9]	Cohort prospective, single-center	32 (93.7%)	65 ± 9	LAD 12, RCA 20, Cx 0	Viability and Ischemia	Yes	CMR	Successful PCI	1, 2, 3	3	Yes
Cardona 2016[10]	Cohort prospective, single-center	32 (75.0%)	59 ± 10	LAD 17, RCA 15, Cx 9	Viability and Ischemia	Yes	CMR	Successful PCI	1, 2, 3	6	Yes
Chen 2019[13]	Cohort prospective, single-center	30 (80.0%)	57 ± 12	LAD 11, RCA 13, Cx 3	Viability	No	CMR	Successful PCI	1	6	Yes
Elias 2017[15]	RCT substudy, multi-center	180 (87.2%)	60 ± 10	LAD 47, RCA 86, Cx 47	Viability	No	CMR	PCI vs. OMT	1	4	Yes
Kiko 2021[22]	Cohort prospective, single-center	15 (86.7%)	70 ± 8	LAD 7, RCA 6, Cx 2	Viability	No	Hybrid ^18^F-FDG PET/CMR	Successful PCI	1	6	No
Kirschbaum 2008[14]	Cohort prospective, single-center	21 (85.7%)	64 ± 11	LAD 11, RCA 8, Cx 2	Viability	No	CMR	Successful PCI	1	5	Yes
Mashayekhi 2018[17]	RCT, single-center	205 (88.3%)	67 ± 9	LAD 40, RCA 129, Cx 36	Viability	No	CMR	PCI vs. OMT	1, 4	6 (12 for clinical)	No
Nakachi 2017[21]	Cohort retrospective, single-center	59 (88.1%)	66 ± 11	LAD 21, RCA 27, Cx 11	Viability	No	CMR	Successful PCI	1	8	No
Obedinskiy 2018[8]	RCT, single-center	94 (83.3%)	57 ± 8	Only RCA	Viability and Ischemia	Yes	CMR	PCI vs. OMT	2, 3, 4	2 (12 for clinical)	Yes
Paul 2011[20]	Cohort prospective, single-center	30 (83.3%)	62 ± 10	LAD 11, RCA 16, Cx 3	Viability	No	CMR	Successful vs. unsuccessful PCI	1	6	No
Pujadas 2013[19]	Cohort prospective, single-center	43 (84%)	64 ± 10	LAD 15, RCA 21, Cx 7	Viability and Ischemia	No	CMR	Successful vs. unsuccessful PCI	1, 2, 3	6	No
Rossello 2016[28]	Cohort prospective, single-center	47 (89%)	62 ± 10	LAD 10, RCA 29, Cx 8	Viability and Ischemia	Yes	CMR	Successful PCI	1, 2, 3	6	Yes
Safley 2011[29]	Cohort retrospective, single-center	301 (68.8%)	65 ± 11	Not defined	Ischemia	No	SPECT or PET	Successful PCI	2	12	No
Schumacher 2019[24]	Cohort prospective, single-center	92 (80%)	62 ± 10	LAD 16, RCA 64, Cx 12	Ischemia	Yes	[^15^O]H_2_O PET	Successful PCI	2	4	No
Schumacher 2020[25]	Cohort prospective, single-center	193 (84%)	63 ± 11	LAD 40, RCA 143, Cx 36	Ischemia	Yes	[^15^O]H_2_O PET	Successful PCI	2	3	Yes
Schumacher 2021[26]	Cohort prospective, single-center	212 (84%)	62 ± 11	LAD 48, RCA 152, Cx 38	Ischemia	Yes	[^15^O]H_2_O PET	Successful PCI	2, 3, 4	3 (2.8 years for clinical)	No
Schumacher 2021[26]	Cohort prospective, single-center	132 (81%)	62 ± 11	LAD 26, RCA 95, Cx 11	Viability	Yes	CMR	Successful PCI	1	3	No
Stuijfzand 2017[11]	Cohort prospective, single-center	69 (86%)	63 ± 10	LAD 14, RCA 51, Cx 4	Viability and Ischemia	Yes	CMR and [^15^O]H_2_O PET	Successful PCI	1, 2	3	Yes
Vitadello 2020[23]	Cohort prospective, multi-center	23 (96%)	61 ± 9	LAD 4, RCA 13, Cx 6	Viability	No	Hybrid ^18^F-FDG PET/CMR	Successful PCI	1	6	No
Zhang 2021[27]	Cohort retrospective, single-center	74 (86.5%)	62 ± 8	Not defined	Ischemia	No	[^15^O]H_2_O PET	Successful PCI	2	6	Yes

*Cx *Circumflex artery,* CMR *Cardiac magnetic resonance,* CTO *Chronic total coronary occlusion,* FDG *Fluorodesoxiglicose,* LAD *Left anterior descending artery,* LV *Left ventricle,* MACE *Major adverse cardiovascular events,* OMT *Optimal medical therapy,* PCI *Percutaneous coronary intervention,* PET *Positron emission tomography,* RCA *Right coronary artery,* SD *Standard deviation,* SPECT *Stress echocardiography, or single-photon emission computed tomography

Functional imaging tests were used prior to PCI to evaluate viability in ten studies (CMR – 8; hybrid ^18^F-FDG PET/CMR – 2), ischemia in five studies ([^15^O]H_2_O PET – 4; SPECT – 1), and both viability and ischemia in six studies (CMR – 5; CMR and [^15^O]H_2_O PET – 1). In nine studies, the presence of ischemia or viability was a predefined inclusion criterion and actively guided the decision to perform PCI. In the remaining twelve studies, these assessments were performed retrospectively, primarily for post hoc subgroup analyses or prognostic evaluation. Notably, among the three RCTs, all documented myocardial viability or ischemia status, but only one required the presence of viability or ischemia as an inclusion criterion, whereas the others enrolled CTO patients irrespective of the result. In the studies using CMR, myocardial segments were considered dysfunctional if SWT was ≤ 45%, and viability was assessed by the transmural extent of infarction (TEI) by late-gadolinium-enhancement (LGE). Dysfunctional segments with a TEI score of ≤ 25% or ≤ 50%, depending on the study criteria, were considered viable. Regarding ^18^F-FDG PET, a segment was defined as viable if the FDG uptake was greater than 50%. The evaluation of ischemic burden varied according to the imaging modality: both CMR and [^15^O]H_2_O PET used criteria based on perfusion defect size and myocardial blood flow quantification, while SPECT assessed the percentage of ischemic myocardium.

The study outcomes varied, with 15 studies assessing LV function, 11 focusing on ischemic burden, six on symptoms and quality of life, and three on MACE. Most studies had follow-up periods between two to six months, with longer follow-ups (≥ 12 months) in studies that assessed clinical event outcomes.

### Risk of bias

**Fig. 2:**
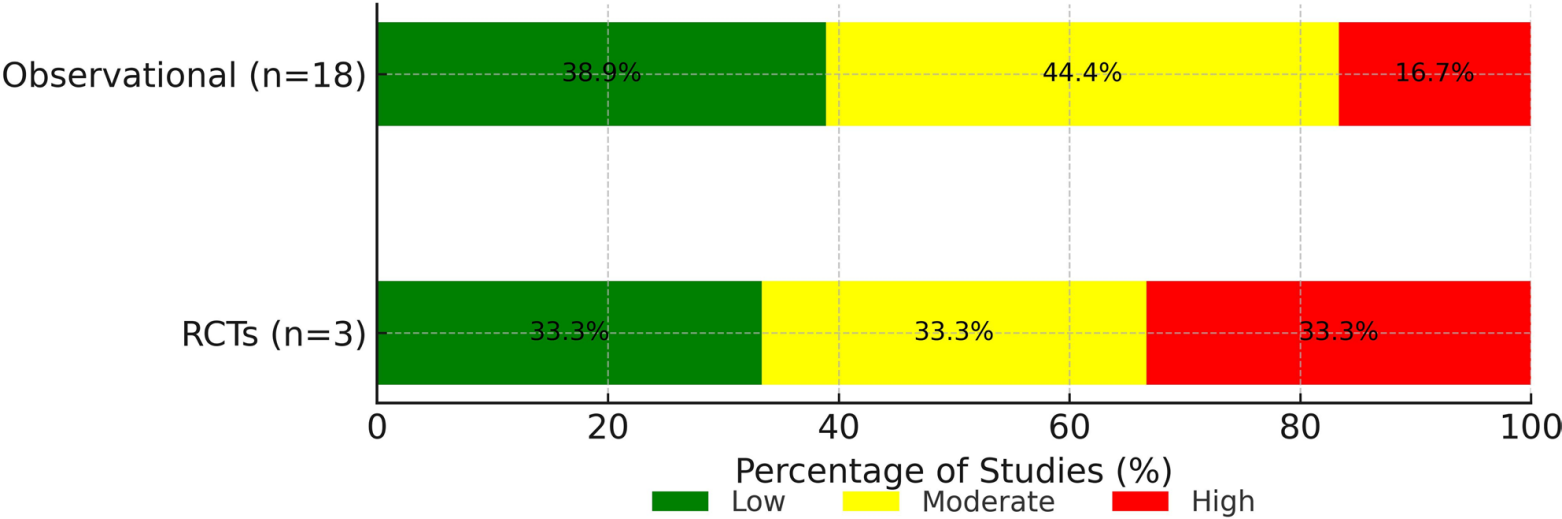
Summary of overall risk of bias in included studies. RCT: Randomized clinical trial

Among the 21 included studies, three were randomized controlled trials assessed using the RoB 2 tool. Of these, one was rated as having low risk of bias, one as having some concerns, and one as high risk. The remaining 18 were observational studies evaluated with the ROBINS-I tool. Of these, three were judged to have a serious overall risk of bias, eight had moderate risk, and seven were considered low risk. The most common sources of bias were related to confounding and missing outcome data. Specifically, confounding was rated as a serious concern in two studies and moderate in 16 studies. Missing outcome data posed a moderate to serious risk in 13 of the 18 non-randomized studies, primarily due to retrospective designs and loss to follow-up. A summary of overall risk of bias for each study is presented in Table 2, and the distribution of risk levels across study designs is shown in Fig. 2. Detailed risk of bias assessments remain available in Supplementary Tables 4 and 5.

**Table 2:** Summary of overall risk of bias

	Design	Assessment Tool	Overall Risk
Elias 2017[15]	RCT	RoB 2	Some concerns
Mashayekhi 2018[17]	RCT	RoB 2	Low
Obendinskiy 2018[8]	RCT	RoB 2	High
Baks 2006[12]	Observational	ROBINS-I	Moderate
Bucciarelli-Ducci 2016[9]	Observational	ROBINS-I	Moderate
Cardona 2016[10]	Observational	ROBINS-I	Low
Chen 2019[13]	Observational	ROBINS-I	Low
Kiko 2021[22]	Observational	ROBINS-I	Moderate
Kirschbaum 2008[14]	Observational	ROBINS-I	Moderate
Nakachi 2017[21]	Observational	ROBINS-I	Low
Paul 2011[20]	Observational	ROBINS-I	Low
Pujadas 2013[19]	Observational	ROBINS-I	Low
Rossello 2016[28]	Observational	ROBINS-I	Moderate
Safley 2011[29]	Observational	ROBINS-I	Serious
Schumacher 2019[24]	Observational	ROBINS-I	Moderate
Schumacher 2020[25]	Observational	ROBINS-I	Low
Schumacher 2021[26]	Observational	ROBINS-I	Low
Schumacher 2021[26]	Observational	ROBINS-I	Moderate
Stuijfzand 2017[11]	Observational	ROBINS-I	Serious
Vitadello 2020[23]	Observational	ROBINS-I	Moderate
Zhang 2021[27]	Observational	ROBINS-I	Serious

*RCT *Randomized clinical trial,* RoB 2* Cochrane Risk-of-Bias 2

### Outcomes

#### LV function and remodelling

- *Global LV Function*

**Fig. 3:**
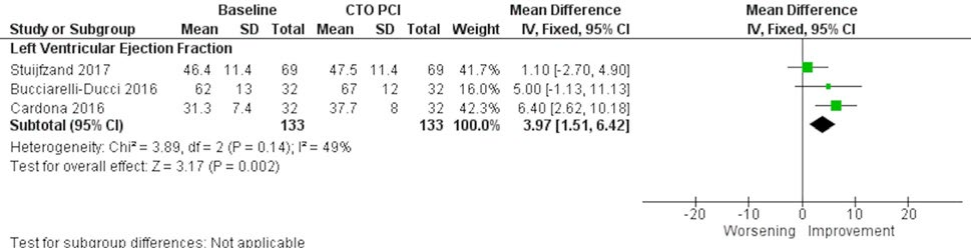
Meta-analysis evaluating the association of successful chronic total occlusion percutaneous revascularization (CTO-PCI) and left ventricular ejection fraction (LVEF) improvement. CTO: Chronic total occlusion; PCI: Percutaneous coronary intervention

**Fig. 4:**
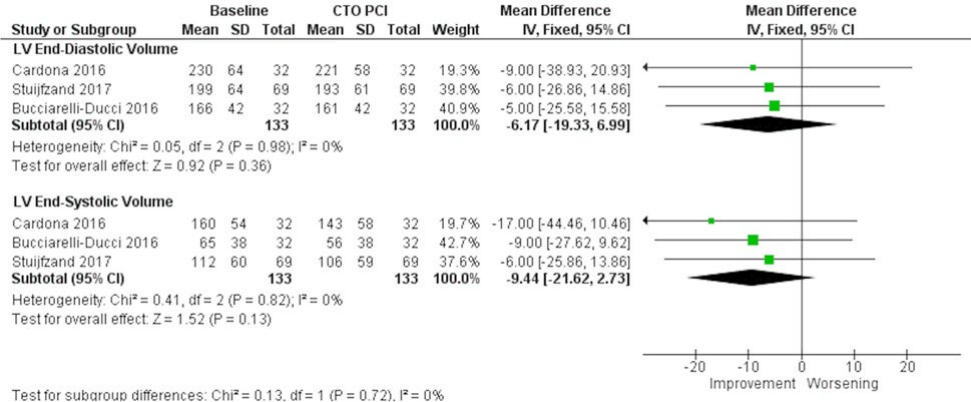
Meta-analysis evaluating the association of successful chronic total occlusion percutaneous revascularization (CTO-PCI) and left ventricular end-diastolic (LVEDV) and end-systolic (LVESV) volumes improvement. CTO: Chronic total occlusion; LV: Left ventricle; PCI: Percutaneous coronary intervention

Three observational studies (133 patients) [9–11] assessed global LV function before and after CTO-PCI, and in all cases, prospective evidence of myocardial viability and/or ischemia—documented by CMR—was an inclusion criterion for the procedure. Meta-analysis revealed that successful PCI was associated with a modest but statistically significant improvement in LVEF (MD: 3.97%; 95% CI: 1.51% to 6.42%; *p* = 0.002; I^2^ = 49%) (Fig. 3), but not with a statistically significant decrease in LVEDV (MD: −6.17 mL; 95% CI: −19.33 mL to 6.99 mL; *p* = 0.36; I^2^ = 0%), and in LVESV (MD: −99.44 mL; 95% CI: −21.62 mL to 2.73 mL; *p* = 0.13; I^2^ = 0%) (Fig. 4). The detailed quality assessment for this and other outcomes can be found in Supplementary Table 6.

b) *Regional LV Function*

**Fig. 5:**
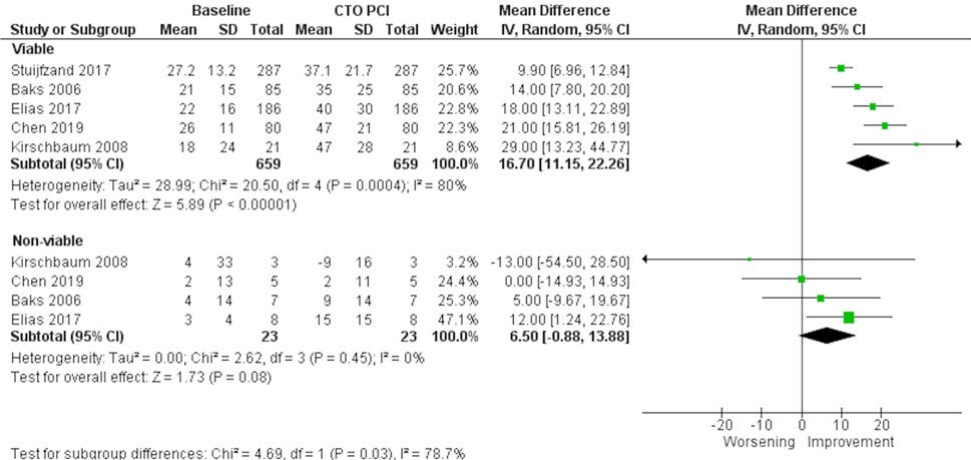
Meta-analysis evaluating the association of successful chronic total occlusion percutaneous revascularization (CTO-PCI) and segmental wall thickness (SWT) in dysfunctional segments, viable or non-viable. CTO: Chronic total occlusion; PCI: Percutaneous coronary intervention

The change in regional SWT in dysfunctional segments supplied by the CTO vessel, comparing viable with no-viable segments, was available in five studies (227 patients; 682 dysfunctional segments), all of which used CMR and included only successful PCI [11–15]. Among these, only Stuijfzand et al. [11] required prospective documentation of viability/ischemia as an inclusion criterion; all other studies assessed viability/ischemia retrospectively. Successful PCI was associated with a significant increase in SWT of viable segments (MD: 16.70%; 95% CI: 11.15% to 22.26%; *p* < 0.001; I^2^ = 80%), but no significant change was observed in non-viable segments (MD: 6.50%; 95% CI: −0.88% to 13.88%; *p* = 0.08; I^2^ = 0%) at the three to six-month follow-up (Fig. 5).

Two sensitivity analyses were performed: (1) Restricting to observational studies only, which yielded results consistent with the main analysis both in viable segments (MD: 16.58%; 95% CI: 9.53% to 23.63%; *p* < 0.01; I² = 83%) and in non-viable segments (MD: 1.62%; 95% CI: −8.53% to 11.76%; *p* = 0.76; I² = 0%) (Supplementary Fig. 6). (2) Excluding Stuijfzand et al. [8], the only study with prospective assessment, which again produced similar results for viable segments (MD: 18.57%; 95% CI: 14.56% to 22.59%; *p* < 0.01; I² = 36%) and for non-viable segments (MD: 6.50%; 95% CI: −0.88% to 13.88%; *p* = 0.08; I² = 0%) (Supplementary Fig. 7). These findings confirm that the inclusion of these studies did not materially influence results.

Among two RCTs comparing PCI to OMT, Elias et al. [15], a sub-study of the EXPLORE trial [16], found significantly greater SWT recovery in dysfunctional segments with TEI < 50% after CTO-PCI (17 ± 27%) versus OMT (11 ± 22%, *p* = 0.02), with no difference in segments with TEI >50%. In contrast, the RCT by Mashayekhi et al. [17], which did not include ischemia or viability as inclusion criteria, found no significant difference in SWT recovery between PCI and OMT groups, even when restricting the analysis to CTO dysfunctional segments, with similar improvement observed in both groups. Restriction of the analysis to the patients with relevant viability in the CTO area also showed no difference in SWT improvement between OMT and PCI groups.

Kirschbaum et al. [14] found no SWT improvement in transmural scars (TEI >75%) at early or 3-year follow-up. In contrast, segments with TEI 25–75% improved significantly at 3 years (39 ± 43%; *p* = 0.04). A TEI cutoff of 25% predicted >10% SWT recovery (OR: 5.63; *p* = 0.01). Schumacher et al. [18] reported SWT normalization in 38% of viable segments post-PCI. Patients with ≥ 2 viable segments (>10% LV) showed greater improvement. In comparisons of successful vs. unsuccessful PCI, Pujadas et al. [19] and Paul et al. [20] found significant SWT recovery only after successful PCI in viable segments.

In the study by Bucciarelli-Ducci et al. [9], regional wall motion improved in 47% of patients with baseline abnormalities, with a significant reduction in the mean visual score (5.9 to 4.5; *p* = 0.003). Cardona et al. [10] found an increase in segments with normal or mild hypokinesia (*p* = 0.001) and a decrease in segments with severe hypokinesia, akinesia, or dyskinesia (*p* = 0.002).

In a study using speckle-tracking echocardiography [21] both longitudinal and circumferential strain improved in segments with TEI ≤ 50%, while no significant change was observed in segments with TEI >50%.

Two studies evaluated viability using hybrid ^18^F-FDG PET/CMR imaging. Kiko et al. [22] found that the greatest wall motion recovery occurred in segments viable on both PET and CMR, with the least improvement in those non-viable on both. There were no significant differences in functional recovery between the PET-viable/CMR-nonviable and PET-nonviable/CMR-viable groups. Vitadello et al. [23] showed that combining PET and CMR improved diagnostic accuracy for viability prediction (AUC = 0.72; *p* = 0.002), surpassing the performance of either modality alone.

#### Ischemic burden

Myocardial perfusion was assessed using [^15^O]H_2_O PET in five studies [11, 24–27], CMR in five [8–10, 19, 28], and SPECT in one [29].

- *PET assessment*

**Fig. 6:**
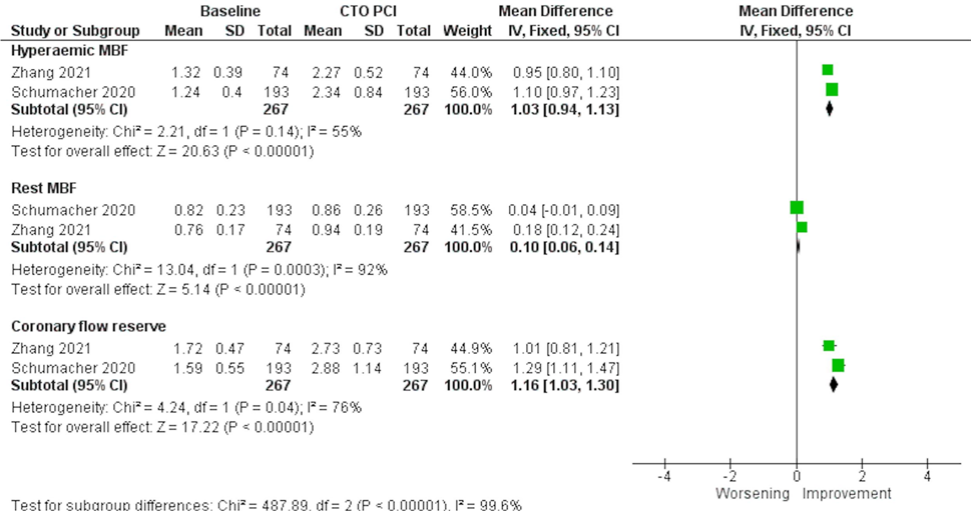
Meta-analysis evaluating the association of successful chronic total occlusion percutaneous revascularization (CTO-PCI) and hyperaemic myocardial blood flow (MBF), rest MBF and coronary flow reserve (CFR). CTO: Chronic total occlusion; MBF: Myocardial blood flow; PCI: Percutaneous coronary intervention

Five studies, including two different populations [25, 27] of 267 patients in total, showed that successful PCI significantly improved hyperaemic MBF (MD: 1.03 mL/min/g; 95% CI: 0.94 mL/min/g to 1.13 mL/min/g; *p* < 0.001; I^2^ = 55%), rest MBF (MD: 0.10 mL/min/g; 95% CI: 0.06 mL/min/g to 0.14 mL/min/g; *p* < 0.001; I^2^ = 92%), and CFR (MD: 1.16; 95% CI: 1.03 to 1.30; *p* < 0.001; I^2^ = 76%) (Fig. 6). This analysis included one study with prospective viability/ischemia assessment and one with retrospective assessment; subgroup analysis was not feasible due to the small number of studies.

Perfusion defect size was significantly reduced in all PET studies. Schumacher et al. [25] found a median decrease of 3 segments [1.5–4.5], varying by initial defect size. ROC analysis identified ≥ 3 segments as the optimal baseline threshold for predicting ≥ 1- or ≥ 2-segment reduction. However, changes in hyperaemic MBF (*p* = 0.75) and CFR (*p* = 0.79) did not differ significantly across groups. These findings were consistent with those presented by Zhang et al. [27].

b) *CMR assessment*

The IMPACTOR-CTO trial [8] found a significantly greater reduction in myocardial ischemia burden (MIB) in the PCI group (13.9 ± 6.1%) than in OMT group (0.3 ± 4.2%; *p* < 0.01). Pujadas et al. [19] reported a significant reduction in ischemia after successful PCI (79% to 30%, *p* < 0.001), whereas ischemia remained essentially unchanged after unsuccessful PCI group (*p* = 0.3). In the remaining three studies that included only successful CTO-PCI, Bucciarelli-Ducci et al. [9] reported visual perfusion score improvement (10.9 to 1.6; *p* < 0.0001), and that myocardial perfusion reserve (MPR) in the CTO territory normalized post-PCI (baseline 1.8 ± 0.7 vs. remote 2.2 ± 0.7; post-PCI: 2.3 ± 0.9; *p* = 0.02). In Cardona’s study [10], there was a significant reduction in the number of segments with an inducible perfusion defect in the CTO territory (0.5 ± 1 vs. 0.2 ± 0.5; *p* = 0.043). Lastly, in the study by Rossello et al. [28], the ischemia burden index (IBI), which measures ischemia using four CMR parameters, decreased by 16 points (19% to 3%; *p* < 0.001).

c) *SPECT assessment*

The only myocardial perfusion assessment using SPECT (and semi-quantitative PET to a lesser extent) was conducted by Safley et al. [29], who used the summed difference score (SDS) to measure ischemia. Among the entire cohort, the mean baseline ischemic burden was 13.1% ± 11.9%, and decreased to 6.9% ± 6.5% following CTO-PCI (*p* < 0.001). Greater improvement occurred in patients with moderate or severe baseline ischemia. Overall, 53.5% patients experienced ≥ 5% reduction post-PCI, with a threshold of ≥ 12.5% baseline ischemia best predicting improvement.

#### Functional capacity and patient-reported outcomes

**Fig. 7:**
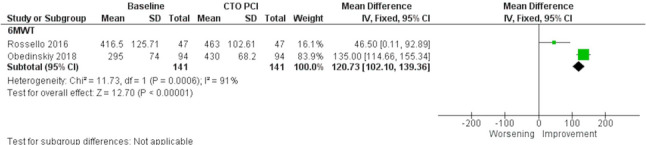
Meta-analysis evaluating the association of successful chronic total occlusion percutaneous revascularization (CTO-PCI) and six-minute walk distance (6MWT). CTO: Chronic total occlusion; PCI: Percutaneous coronary intervention; 6MWT: Six-Minute Walk Test

Two studies (*n* = 141 patients) [8, 28], both requiring prospective CMR evidence of viability or ischemia before PCI, evaluated changes in physical capacity using the 6MWT. The distance significantly increased after successful PCI (MD: 120.73 m; 95% CI: 102.10 m to 139.36 m; *p* < 0.001; I^2^ = 91%) (Fig. 7). The IMPACTOR-CTO Trial showed no significant improvement in the OMT group. Additionally, Obedinskiy et al. found improvements in all domains of the Short Form-36 Health Survey (SF-36) after PCI, whereas no improvement was noted in the OMT group.

In the study by Bucciarelli-Ducci et al. [9], which included patients with evidence of viability and/or ischemia, the total SAQ score increased from 54 to 89 (*p* < 0.001) with improvements across subscales. Cardona et al. [10], with similar inclusion criteria, noted fewer patients reporting angina (34.4% to 3.1%; *p* = 0.002), BNP reduction (323 to 123 pg/ml; *p* = 0.004), and better NYHA class at follow-up (72% to 100% in class I and II; *p* = 0.004).

The role of residual ischemia post-CTO-PCI was assessed by Schumacher et al. [26]. Patients with residual hyperaemic MBF >2.3 ml/min/g in the CTO area were more likely to be free from angina and exertional dyspnea at long-term follow-up, compared to patients with lower residual MBF levels, despite of the use of a higher number of anti-anginal agents in the latter group. However, long-term freedom from angina or dyspnea did not differ significantly according to the presence or absence of ≥ 3 segments perfusion defect reduction and residual perfusion defect.

#### Clinical outcomes

The prognostic impact of CTO-PCI was analyzed in two RCTs. The REVASC Trial [17], although involving only 77% of patients with relevant viability in the CTO area, reported lower 12-month MACE with PCI versus OMT. This benefit was mainly driven by a reduced need for clinically driven revascularization, with no difference in fatal outcomes between the groups. On the other hand, the IMPACTOR-CTO Trial [8] found no difference in MACE-free survival between the PCI and OMT groups at 12 months (94.9% vs. 100%; *p* = 0.19).

In the [^15^O]H_2_O PET cohort from Schumacher et al. [26], with a median follow-up of 2.8 years and focusing solely on successful CTO-PCI cases, patients with ≥ 3 segments defect reduction or above-median hyperaemic MBF post-PCI had improved event-free survival. Better outcomes were also observed in patients without residual defects or with residual hyperaemic MBF >2.3 ml/min/g in the CTO area.

Safley et al. [29], showed that patients with ≥ 5% ischemia reduction had lower 12-month MACE (18.6% vs. 28.6%; *p* = 0.042), mainly due to reduced revascularization (14.3% vs. 25.0%; *p* = 0.019). There was no difference in 12-month mortality (0.6% vs. 1.4%, *p* = 0.60), but Kaplan–Meier analysis showed a survival advantage for patients with reduced ischemia following CTO-PCI when followed for up to 6 years.

#### Certainty of evidence

The certainty of evidence for the primary outcomes was assessed using the GRADE framework (Table 3). Overall, the certainty ranged from moderate to low. Evidence supporting improvements in regional LV function (SWT), ischemic burden, and symptoms/quality of life was rated as moderate, reflecting consistent effects across studies despite small sample sizes. By contrast, certainty was rated as low for global LV function and MACE, mainly due to the predominance of observational designs, limited sample sizes, heterogeneity in imaging modalities, and short follow-up durations. These limitations reduce confidence in the estimated effects and underscore the need for large, well-designed randomized trials.

**Table 3:** GRADE summary of findings

Outcome	No. of studies (designs)	Effect (summary)	Certainty of evidence (GRADE)	Comments
Global LV function: LVEF	3 obs.	↑ LVEF by ~ 3.9%	⬤⬤◯◯ Low	Small cohorts, observational only; all prospective viability/ischemia inclusion.
Global LV function: LVEDV, LVESV	3 obs.	No significant change	⬤⬤◯◯ Low	Consistent null effect; limited by small size and observational design.
Regional LV function: SWT in viable segments	2 RCT + 7 obs.	SWT improved in viable segments only (MD ~ 16.7%) vs. no effect in non-viable segments Mashayekhi et al. found no difference vs. OMT. Improvement seen only in successful PCI vs. failed PCI.	⬤⬤⬤◯ Moderate	Effect consistent across studies, but mostly observational; high heterogeneity; mixed RCT findings.
Ischemic burden	1 RCT + 10 obs.	↓ Perfusion defect size, ↑ Hyperaemic MBF, ↑ CFR	⬤⬤⬤◯ Moderate	Consistent benefit across imaging modalities; heterogeneity in methodology; limited RCT data
Symptoms and Quality of Life	1 RCT + 4 obs.	↑ 6MWT (+ 120 m), ↑ SAQ, ↑ SF-36 scores, ↓ BNP	⬤⬤⬤◯ Moderate	Improvement exceeds MCID; small number of studies; consistent improvements but risk of bias and imprecision.
MACE	2 RCTs + 2 obs.	Mixed results; some ↓ repeat revascularization, no consistent mortality benefit	⬤⬤◯◯ Low	Underpowered; conflicting results across RCTs.

* BNP* B-type natriuretic peptide, *CFR* Coronary flow reserve, *LVEF* Left ventricular ejection fraction, *LVEDV* Left ventricular end-diastolic volume, *LVESV* Left ventricular end-systolic volume, *MACE* Major adverse cardiovascular events, *MBF* Myocardial blood flow, *MCID* Minimum clinically important difference, *MD* Mean difference, *Obs* Observational, *PCI* Percutaneous coronary intervention, *RCT* Randomized clinical trial, *SAQ* Seattle angina questionnaire, *SF-36* Questionnaire short form 36 health survey, *SWT* Systolic wall thickening, *6MWT* Six-minute walk test

GRADE certainty of evidence: ⬤⬤⬤⬤ High; ⬤⬤⬤◯ Moderate; ⬤⬤◯◯ Low; ⬤◯◯◯ Very low

### Reporting bias

Funnel plots were constructed for all meta-analysed outcomes (Supplementary Figs. 1–5). Visual inspection did not show clear evidence of asymmetry. However, these findings should be interpreted with caution because each analysis included only 2–5 studies, which precludes meaningful application of formal tests such as Egger’s or Begg’s. Consequently, the possibility of small-study effects cannot be excluded.

## Discussion

Our systematic review provides an extensive evaluation of the impact of ischemia and/or viability documentation prior to CTO-PCI on patient outcomes, supporting a more tailored approach to CTO revascularization. Five key findings emerged: (1) Successful CTO-PCI was associated with modest but significant improvements in LVEF, although no consistent reductions in LV volumes were observed; (2) Regional functional recovery was closely associated with myocardial viability, with significant SWT improvement confined to viable segments; (3) CTO-PCI consistently reduced ischemic burden, with larger baseline perfusion defects predicting greater improvement; (4) Patients achieving extensive ischemic burden reduction and no residual ischemia after CTO-PCI, as assessed by quantitative measures of MBF, were more likely to remain free of angina and exertional dyspnea and had lower rates of all-cause death and nonfatal MI; (5) Successful CTO-PCI with documented previous ischemia and/or viability resulted in significant improvements in 6MWT distance, an effect not observed in the OMT-only group. These pooled results should, however, be interpreted cautiously given the substantial statistical heterogeneity across several outcomes, likely reflecting variability in study design, imaging modalities, and follow-up durations.

### LV function and remodeling

The impact of successful CTO-PCI on LV function, including LVEF and LV reverse remodeling, has been controversial. Our analyses, restricted to patients with documented viability and/or ischemia in the territory supplied by the CTO, showed that PCI was associated with a slight but significant increase in LVEF, although no significant reduction in LV volumes was observed. The modest improvement in LVEF likely reflects the biological limits of global reverse remodeling in CTO patients, with most recovery occurring regionally in viable segments. Variability in baseline LVEF, short follow-up, and methodological differences across studies may also have contributed. Although the absolute improvement in LVEF of approximately 4% may appear modest, its clinical significance should not be underestimated. Evidence from large clinical trials, such as the STICH Trial [30], has shown that while a ≥ 10% increase in LVEF at 24 months is independently associated with reduced mortality, smaller improvements in LVEF also trend towards better outcomes, including lower rates of heart failure decompensation and improved survival. These findings suggest that even modest gains in LVEF after CTO-PCI may translate into meaningful clinical benefits, especially in patients with impaired baseline ventricular function.

Regional functional recovery was more evident than global improvement. Only viable myocardial segments demonstrated significant SWT recovery after successful CTO-PCI. The benefit was less apparent in non-viable segments and absent when PCI was unsuccessful [19, 20], further emphasizing the importance of successful recanalization and careful patient selection. Most studies used CMR as the functional imaging test to evaluate viability, but two [22, 23] used both CMR and ^18^F-FDG PET with good agreement between imaging data, achieving the highest functional recovery of wall motion abnormalities in the PET-viable/CMR-viable group, in comparison with PET or CMR alone. Overall, CMR emerged as the predominant and reproducible modality for assessing viability and regional function, while PET offered complementary metabolic information. The results presented reinforce that CTO revascularization can reverse regional myocardial dysfunction of hibernating myocardium and support viability-guided revascularization strategies. However, a slight improvement was also observed with OMT in some studies, suggesting that collateral-mediated perfusion may play a modest role.

To test the robustness of our findings, we performed two sensitivity analyses. First, we restricted the analysis to observational studies only, yielding results consistent with the primary analysis. Second, we repeated the analysis after excluding the only study that required prospective documentation of viability or ischemia, keeping only those that assessed these parameters retrospectively; the effect size remained essentially unchanged. These findings confirm that the observed benefit was not driven by study design or by selection criteria. Nevertheless, the predominance of observational studies and retrospective assessments limits firm conclusions about true viability-guided PCI. While improvements in SWT reflect meaningful recovery of regional contractility, direct evidence that this translates into better survival or reduced clinical events is still limited. Further studies are needed to explore whether these regional improvements translate into clinically meaningful outcomes and to define the incremental prognostic value of multimodality viability and ischemia testing.

### Ischemia and myocardial perfusion assessment

Accurate identification of jeopardized ischemic myocardial areas is critical for optimizing blood flow restoration. Non-invasive assessment of ischemia can be performed via visual assessment of regional perfusion defects. However, quantitative MBF measures allow further risk stratification by enabling assessment of individuals with known or suspected diffusely impaired MBF, e.g. with multivessel disease or microvascular dysfunction [5]. Our analysis, focusing on quantitative PET measures, found that successful CTO-PCI was associated with significant increases in hyperaemic MBF and CFR, with a smaller but significant increase in rest MBF. Semi-quantitative PET assessment also demonstrated significant reductions in perfusion defect size across all studies [11, 24–27], with greater reductions observed in patients with larger baseline perfusion defects. Similar benefits were observed with CMR-based assessments [9, 28], confirming that ischemia reduction was significantly greater in PCI-treated patients than in those treated with OMT [8]. In contrast, patients with failed PCI did not experience a meaningful reduction in ischemia [19]. In the only study using SPECT [29], semi-quantitative analysis also found significant reductions in ischemic burden among patients with moderate or severe baseline ischemia. Taken together, these data support quantification of ischemia as a key tool for guiding revascularization, suggesting that successful PCI should be judged not only by angiographic recanalization but also by physiological resolution of ischemia. In this regard, PET was the predominant and most reproducible modality for ischemia quantification in our review, offering robust prognostic information through MBF and CFR. CMR, while mainly used for viability assessment, also provided valuable quantitative and semi-quantitative perfusion data. By contrast, SPECT was less precise, though more widely available. These findings highlight that PET and CMR play complementary roles, while hybrid or multimodality approaches may further improve patient selection for CTO-PCI.

### Symptom improvement and quality of life

The primary goal of CTO recanalization is to alleviate exercise-limiting symptoms and improve quality of life [1]. Our analysis revealed a significant improvement in physical activity performance, as measured by the 6MWT, in patients with documented ischemia and/or viability undergoing successful CTO-PCI. This improvement was not observed in the OMT group in the IMPACTOR-CTO trial [8]. The observed gains in 6MWT distance with CTO-PCI exceed the minimal clinically important difference commonly reported for heart failure populations, which is approximately 30–32 m [31], reinforcing the interpretation of this effect as a meaningful functional improvement.

Consistent with functional improvement, patient-reported outcomes also demonstrated substantial improvements. The SAQ showed marked gains in angina frequency, physical limitation, and quality-of-life domains, reflecting reduced symptom burden and better daily functioning. Similarly, the SF-36 captured broader improvements in physical, emotional, and social well-being, underscoring that the benefits of CTO-PCI extend beyond disease-specific measures to overall health status. Improvements in NYHA functional class and reductions in BNP levels were also reported, further supporting enhanced symptomatic relief and functional capacity.

Importantly, Schumacher et al. [26] found that patients with greater ischemia reduction, as measured by hyperaemic MBF, were more likely to be free from angina and dyspnea on exertion at long-term follow-up. These findings reinforce the link between physiological improvements and subjective outcomes and advocate for a tailored, ischemia-guided revascularization approach.

### Clinical outcomes

The impact of CTO-PCI on clinical outcomes has yielded mixed results. RCTs such as REVASC [17] and IMPACTOR-CTO [8] demonstrated functional and quality-of-life improvements but no clear survival benefit, whereas observational studies suggested lower mortality and event rates among patients who achieved ischemia reduction. In particular, residual ischemia after CTO-PCI was associated with worse outcomes. Nevertheless, these findings derive predominantly from observational cohorts with heterogeneous endpoint definitions and limited follow-up durations, which restrict causal inference. Although encouraging, the available data remain underpowered to assess mortality or MACE with certainty. Accordingly, the prognostic associations observed should be regarded as exploratory and hypothesis-generating rather than confirmatory. While ischemia reduction appears to correlate with improved outcomes, the evidence remains limited, and any prognostic benefit of CTO-PCI should be interpreted with caution until validated by adequately powered randomized trials.

### Guidelines and ongoing trials

Our findings may offer an additional perspective to current recommendations. While guidelines remain cautious due to the neutral results of RCTs, the trends we observed – suggesting improvements in LV function, ischemic burden, and quality of life in patients with documented ischemia or viability - highlight the potential importance of careful patient selection. These observations could help explain some of the differences between guideline positions, as most prior studies did not systematically incorporate viability or ischemia testing.

Ongoing randomized trials are expected to provide critical evidence. The ISCHEMIA-CTO trial (NCT03563417) is specifically evaluating whether CTO-PCI improves quality of life and reduces MACE in patients with significant ischemia, thereby addressing the role of ischemia-guided strategies. The NOBLE-CTO trial (NCT03392415) is designed to clarify the broader prognostic impact of CTO revascularization compared with medical therapy, while not requiring systematic viability/ischemia testing in all cases. Until such data become available, current recommendations remain based on limited evidence, underscoring the importance of individualized decision-making guided by ischemia/viability assessment and patient-reported symptoms. Consistent with this, our GRADE assessment indicated that the overall certainty of evidence supporting these findings was moderate to low, further reinforcing the need for cautious interpretation and confirmation in future randomized trials.

In the broader context of clinical practice, our findings highlight the potential role of ischemia and viability assessment in selecting patients most likely to benefit from CTO-PCI. By supporting a more individualized approach to revascularization based on each patient’s ischemic and functional profile, these results may, in time, help guide future guideline refinements as ongoing randomized evidence becomes available.

## Limitations

This study has the inherent limitations of systematic reviews and meta-analyses. The optimal design to address our research question would be a RCT requiring documented ischemia or viability before enrollment. Among the included studies, only three were RCTs, and only one (Obedinskiy et al. [8]) mandated the confirmation of ischemia or viability as an inclusion criterion; the others documented these parameters without requiring them for enrollment. The remaining studies were observational, which, despite being of generally good quality, cannot replace large-scale RCTs and increase the risk of residual confounding. Because treatment allocation in these studies was based on clinical judgment rather than randomization, confounding by indication cannot be excluded, which may have biased outcomes in favor of PCI.

The heterogeneity in study designs adds further complexity. RCTs primarily compared CTO-PCI to OMT, whereas observational studies evaluated successful PCI or compared successful vs. failed PCI. Importantly, only one RCT contributed data to the SWT meta-analysis and one to the 6MWT outcome, limiting the feasibility of formal subgroup or meta-regression analysis by study design. To address this, we performed a sensitivity analysis: (i) excluding the only study with prospective viability assessment in the SWT meta-analysis, and (ii) restricting SWT analyses to observational studies. Both yielded results consistent with the main analysis, supporting the robustness of our findings.

Heterogeneity was also present in imaging modalities. Although we performed separate analyses for CMR-based and PET-based outcomes, and these subgroupings reduce modality-related confounding, direct comparisons across imaging techniques were not feasible. Moreover, only nine studies required ischemia/viability prospectively as part of PCI decision-making, while the remainder assessed these parameters retrospectively. This variation, combined with the lack of uniform assessment methods and standardized reporting, limits the strength of conclusions regarding viability- or ischemia-guided CTO-PCI in routine practice.

The study by Mashayekhi et al. [17], despite meeting initial inclusion criteria, could not be included in quantitative analyses. CTO inclusion in that trial was not based on prior viability or perfusion documentation, and outcomes were not reported separately for patients with confirmed ischemia/viability. Specifically, LVEF, LV volumes, and SWT were not stratified by viable versus non-viable segments. As our analyses focused exclusively on patients with documented ischemia/viability, aggregated data could not be used, and additional data could not be obtained from the authors.

Some outcomes also showed substantial statistical heterogeneity (e.g.,, SWT among viable segments, rest MBF, and 6MWT), as indicated by high I² values. While random-effects models were applied to account for between-study variability, the small number of available studies precluded more detailed subgroup or meta-regression analyses. Similarly, the limited number of studies contributing to each outcome restricted the ability to formally assess publication bias using tests such as Egger’s or Begg’s. Although we performed visual inspection of funnel plots, the possibility of small-study effects cannot be definitively excluded.

Finally, follow-up duration varied across studies, most spanning between two to six months. Longer follow-up was largely limited to clinical event endpoints, and data on MACE were derived from only a few studies, with heterogeneous definitions and short observation periods. As a result, these analyses were underpowered, and the long-term prognostic impact of CTO-PCI remains uncertain. Future adequately powered studies with extended follow-up are needed, particularly in patients selected based on ischemia or viability testing.

Taken together, the predominance of observational data, along with heterogeneity in study design, imaging modalities, endpoints, and follow-up duration, limits the certainty and generalizability of the pooled findings. Consequently, the observed associations should be regarded as hypothesis-generating rather than confirmatory.

## Conclusions

This comprehensive review suggests that ischemia and viability assessment may help guide CTO-PCI decision-making and identify patients most likely to derive benefit. Successful CTO-PCI is associated with improved LV regional wall motion when viable myocardium is present, although the clinical significance of regional SWT improvement requires further investigation. CTO recanalization also appears to reduce ischemic burden, but the extent to which ischemia reduction translates into long-term clinical benefit remains uncertain given the predominance of observational data and study heterogeneity.

Future research should focus on defining the optimal imaging modalities and quantitative thresholds for ischemia and viability that best predict recovery and outcomes. Ongoing randomized trials, such as ISCHEMIA-CTO and NOBLE-CTO, are expected to provide critical evidence regarding the long-term prognostic impact of ischemia-guided CTO-PCI and help refine patient selection strategies in clinical practice.

## Supplementary Information

Supplementary Material 1.

Supplementary Material 2.

Supplementary Material 3.

Supplementary Material 4.

Supplementary Material 5.

Supplementary Material 6.

Supplementary Material 7.

Supplementary Material 8.

## Data Availability

The datasets used and/or analyzed during the current study are available from the corresponding author on reasonable request.

## Abbreviations

PCI: Percutaneous coronary intervention
CTO: Chronic total occlusions
LV: Left ventricular
TIMI: Thrombolysis in myocardial infarction
RCTs: Randomized clinical trials
OMT: Optimal medical therapy
MACE: Major adverse cardiovascular events
PRISMA: Preferred reporting items for systematic reviews and meta-Analyses
CMR: Cardiac magnetic resonance
PET: Positron emission tomography
SPECT: Single-photon emission computed tomography
CENTRAL: Cochrane central register of controlled trials
MeSH: Medical subject headings
SWT: Systolic wall thickening
LVEF: Left ventricular ejection fraction
LVEDV: Left ventricular end-diastolic volume
LVESV: Left ventricular end-systolic volume
6MWT: Six-minute walk test
SAQ: Seattle angina questionnaire
SF: 36-Questionnaire short form 36 health survey
NYHA: New York heart association
MI: Myocardial infarction
GRADE: Grading recommendations assessment, development and evaluation
MD: Mean differences
CIs: Confidence intervals
RCA: Right coronary artery
TEI: Transmural extent of infarction
LGE: Late-gadolinium-enhancement
OR: Odds ratio
MBF: Myocardial blood flow
CFR: Coronary flow reserve
ROC: Receiver operating characteristic
MIB: Myocardial ischemia burden
MPR: Myocardial perfusion reserve
IBI: Ischemia burden index
ACC/AHA/SCAI: American college of cardiology/American heart association/society for cardiovascular angiography and interventions
EAPCI: European association of percutaneous cardiovascular interventions
ESC: European society of cardiology
EACVI: European association of cardiovascular imaging
CCTA: Coronary computed tomographic angiography
