# Supplementary figures and images for: Effect of percutaneous coronary intervention on chronic total occlusions with documented viability or ischemia: a systematic review and meta-analysis
Source: BMC Cardiovasc Disord. 2025 Dec 4;26:17. doi: 10.1186/s12872-025-05405-0 (PMC12781582; doi:10.1186/s12872-025-05405-0)

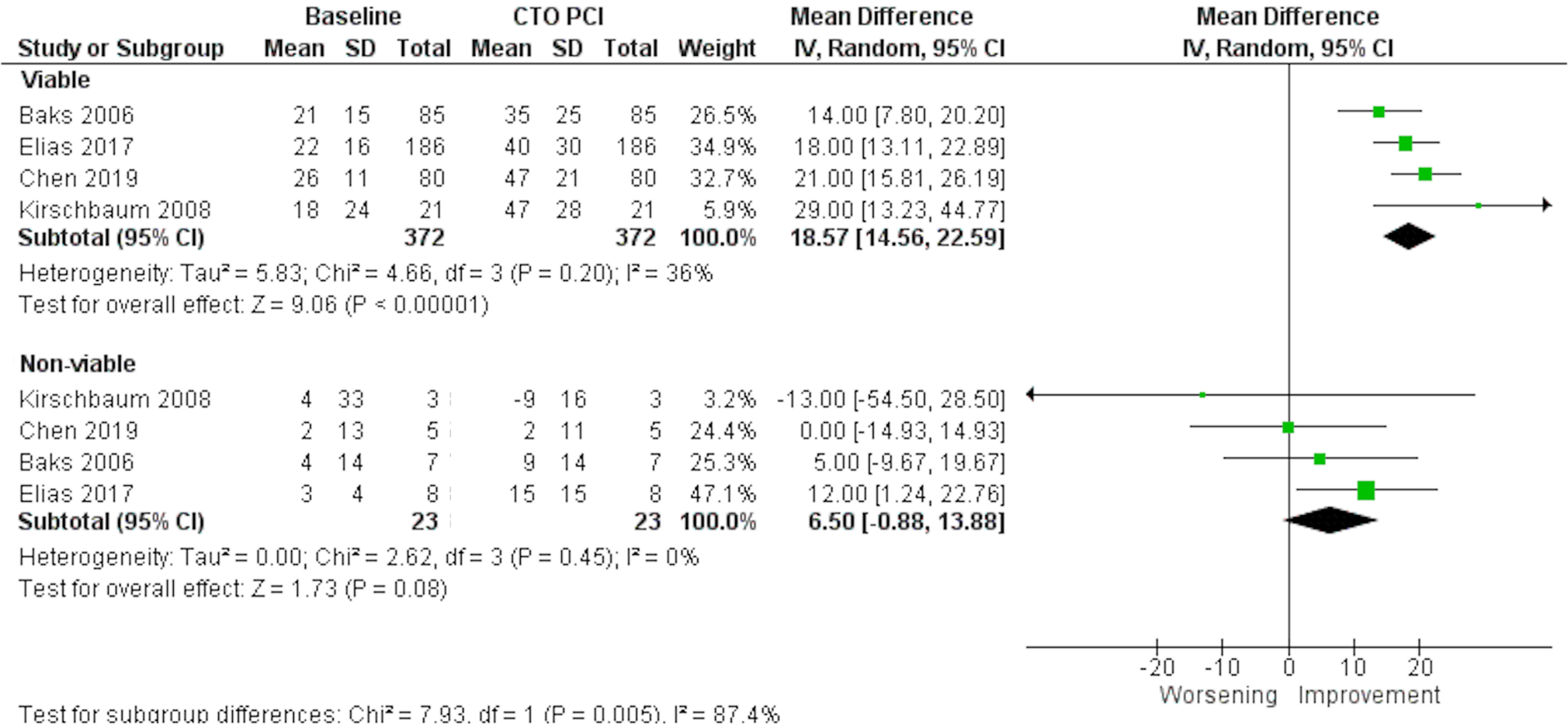

Supplement: Supplementary file 1 — Supplementary Material 1. [file 12872_2025_5405_MOESM1_ESM.tiff]

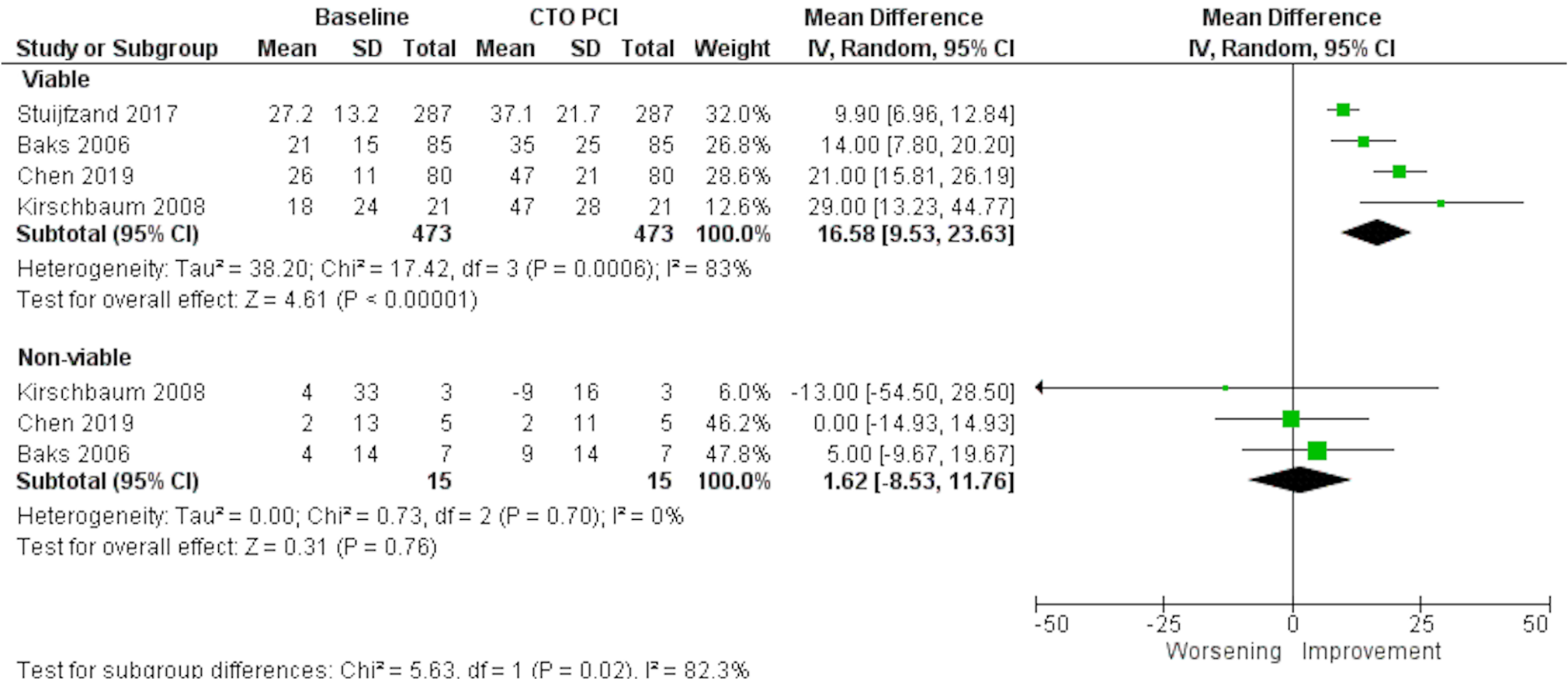

Supplement: Supplementary file 2 — Supplementary Material 2. [file 12872_2025_5405_MOESM2_ESM.tiff]

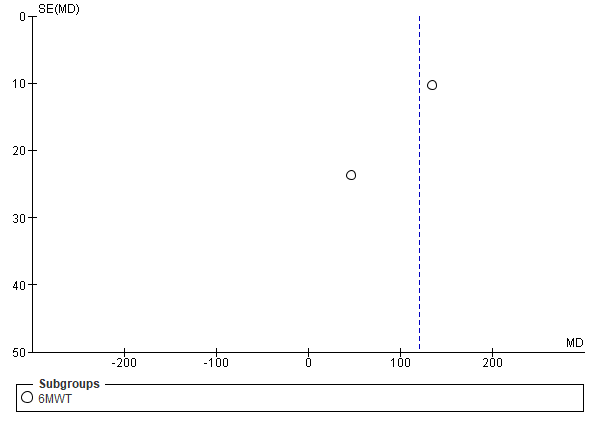

Supplement: Supplementary file 4 — Supplementary Material 4. [file 12872_2025_5405_MOESM4_ESM.tiff]

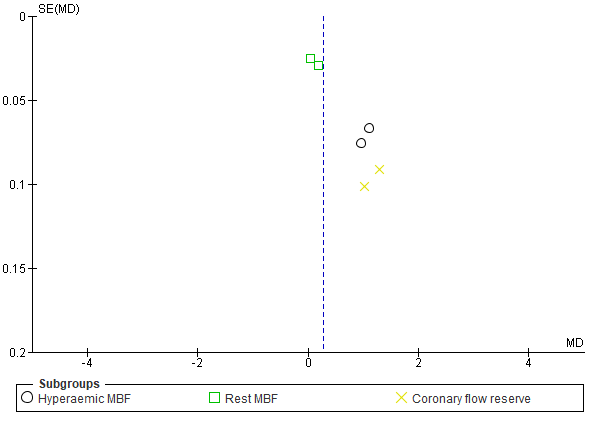

Supplement: Supplementary file 5 — Supplementary Material 5. [file 12872_2025_5405_MOESM5_ESM.tiff]

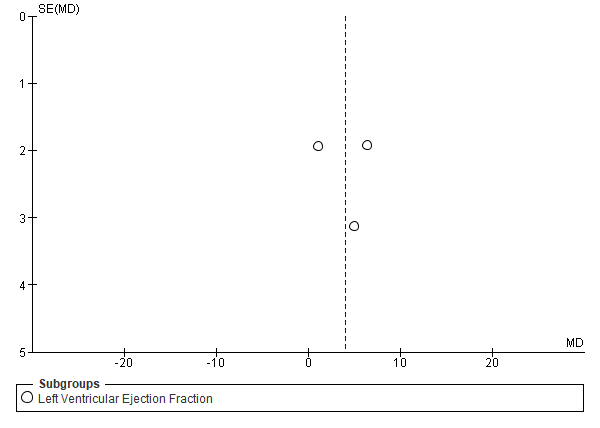

Supplement: Supplementary file 6 — Supplementary Material 6. [file 12872_2025_5405_MOESM6_ESM.tiff]

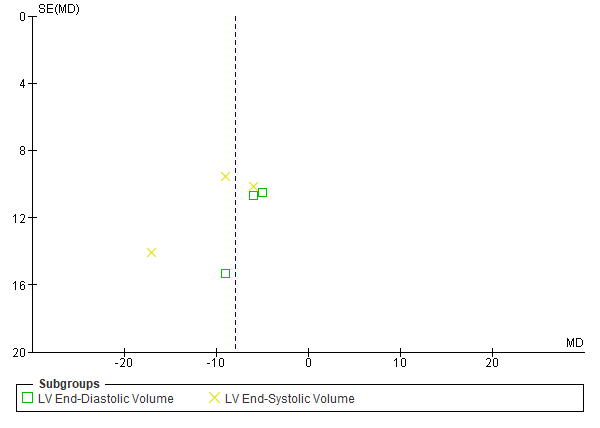

Supplement: Supplementary file 7 — Supplementary Material 7. [file 12872_2025_5405_MOESM7_ESM.tiff]

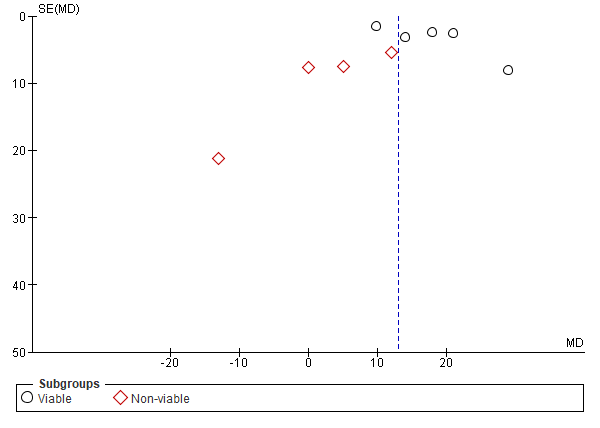

Supplement: Supplementary file 8 — Supplementary Material 8. [file 12872_2025_5405_MOESM8_ESM.tiff]
