# Supplementary material for: Effect of percutaneous coronary intervention on chronic total occlusions with documented viability or ischemia: a systematic review and meta-analysis
Source: BMC Cardiovasc Disord. 2025 Dec 4;26:17. doi: 10.1186/s12872-025-05405-0 (PMC12781582; doi:10.1186/s12872-025-05405-0)
Supplement: Supplementary file 3 — Supplementary Material 3. [file 12872_2025_5405_MOESM3_ESM.docx]

**Supplementary data**

**Supplementary Table 1.** Preferred Reporting Items for Systematic Reviews and Meta-Analyses (PRISMA)

| **Section and Topic** | **Item #** | **Checklist item** | **Location where item is reported** |
| --- | --- | --- | --- |
| **TITLE** | | |  |
| Title | 1 | Identify the report as a systematic review. | 1 |
| **ABSTRACT** | | |  |
| Abstract | 2 | See the PRISMA 2020 for Abstracts checklist. | 3 |
| **INTRODUCTION** | | |  |
| Rationale | 3 | Describe the rationale for the review in the context of existing knowledge. | 5 |
| Objectives | 4 | Provide an explicit statement of the objective(s) or question(s) the review addresses. | 5 |
| **METHODS** | | |  |
| Eligibility criteria | 5 | Specify the inclusion and exclusion criteria for the review and how studies were grouped for the syntheses. | 6 |
| Information sources | 6 | Specify all databases, registers, websites, organisations, reference lists and other sources searched or consulted to identify studies. Specify the date when each source was last searched or consulted. | 6-7 |
| Search strategy | 7 | Present the full search strategies for all databases, registers and websites, including any filters and limits used. | 7 |
| Selection process | 8 | Specify the methods used to decide whether a study met the inclusion criteria of the review, including how many reviewers screened each record and each report retrieved, whether they worked independently, and if applicable, details of automation tools used in the process. | 7 |
| Data collection process | 9 | Specify the methods used to collect data from reports, including how many reviewers collected data from each report, whether they worked independently, any processes for obtaining or confirming data from study investigators, and if applicable, details of automation tools used in the process. | 7-8 |
| Data items | 10a | List and define all outcomes for which data were sought. Specify whether all results that were compatible with each outcome domain in each study were sought (e.g. for all measures, time points, analyses), and if not, the methods used to decide which results to collect. | 8 |
|  | 10b | List and define all other variables for which data were sought (e.g. participant and intervention characteristics, funding sources). Describe any assumptions made about any missing or unclear information. | 8 |
| Study risk of bias assessment | 11 | Specify the methods used to assess risk of bias in the included studies, including details of the tool(s) used, how many reviewers assessed each study and whether they worked independently, and if applicable, details of automation tools used in the process. | 8-9 |
| Effect measures | 12 | Specify for each outcome the effect measure(s) (e.g. risk ratio, mean difference) used in the synthesis or presentation of results. | 9 |
| Synthesis methods | 13a | Describe the processes used to decide which studies were eligible for each synthesis (e.g. tabulating the study intervention characteristics and comparing against the planned groups for each synthesis (item #5)). | 9 |
|  | 13b | Describe any methods required to prepare the data for presentation or synthesis, such as handling of missing summary statistics, or data conversions. | 9 |
|  | 13c | Describe any methods used to tabulate or visually display results of individual studies and syntheses. | 9 |
|  | 13d | Describe any methods used to synthesize results and provide a rationale for the choice(s). If meta-analysis was performed, describe the model(s), method(s) to identify the presence and extent of statistical heterogeneity, and software package(s) used. | 9 |
|  | 13e | Describe any methods used to explore possible causes of heterogeneity among study results (e.g. subgroup analysis, meta-regression). | NA |
|  | 13f | Describe any sensitivity analyses conducted to assess robustness of the synthesized results. | NA |
| Reporting bias assessment | 14 | Describe any methods used to assess risk of bias due to missing results in a synthesis (arising from reporting biases). | 10 |
| Certainty assessment | 15 | Describe any methods used to assess certainty (or confidence) in the body of evidence for an outcome. | 10 |
| **RESULTS** | | |  |
| Study selection | 16a | Describe the results of the search and selection process, from the number of records identified in the search to the number of studies included in the review, ideally using a flow diagram. | 10-11 |
|  | 16b | Cite studies that might appear to meet the inclusion criteria, but which were excluded, and explain why they were excluded. | 10-11 |
| Study characteristics | 17 | Cite each included study and present its characteristics. | 12-13 |
| Risk of bias in studies | 18 | Present assessments of risk of bias for each included study. | 13-14 |
| Results of individual studies | 19 | For all outcomes, present, for each study: (a) summary statistics for each group (where appropriate) and (b) an effect estimate and its precision (e.g. confidence/credible interval), ideally using structured tables or plots. | 14-22 |
| Results of syntheses | 20a | For each synthesis, briefly summarise the characteristics and risk of bias among contributing studies. | 14-22 |
|  | 20b | Present results of all statistical syntheses conducted. If meta-analysis was done, present for each the summary estimate and its precision (e.g. confidence/credible interval) and measures of statistical heterogeneity. If comparing groups, describe the direction of the effect. | 14-22 |
|  | 20c | Present results of all investigations of possible causes of heterogeneity among study results. | 14-22 |
|  | 20d | Present results of all sensitivity analyses conducted to assess the robustness of the synthesized results. | 16-17 |
| Reporting biases | 21 | Present assessments of risk of bias due to missing results (arising from reporting biases) for each synthesis assessed. | 23 |
| Certainty of evidence | 22 | Present assessments of certainty (or confidence) in the body of evidence for each outcome assessed. | 22-23 |
| **DISCUSSION** | | |  |
| Discussion | 23a | Provide a general interpretation of the results in the context of other evidence. | 23-28 |
|  | 23b | Discuss any limitations of the evidence included in the review. | 29-31 |
|  | 23c | Discuss any limitations of the review processes used. | 29-31 |
|  | 23d | Discuss implications of the results for practice, policy, and future research. | 29-31 |
| **OTHER INFORMATION** | | |  |
| Registration and protocol | 24a | Provide registration information for the review, including register name and registration number, or state that the review was not registered. | 6 |
|  | 24b | Indicate where the review protocol can be accessed, or state that a protocol was not prepared. | 6 |
|  | 24c | Describe and explain any amendments to information provided at registration or in the protocol. | 6 |
| Support | 25 | Describe sources of financial or non-financial support for the review, and the role of the funders or sponsors in the review. | 2 |
| Competing interests | 26 | Declare any competing interests of review authors. | 1 |
| Availability of data, code and other materials | 27 | Report which of the following are publicly available and where they can be found: template data collection forms; data extracted from included studies; data used for all analyses; analytic code; any other materials used in the review. | NA |

**Supplementary Table 2.** PICO (Population, Intervention, Comparator, Outcome) description

| Abbreviation | PICO Elements |
| --- | --- |
| P (Population) | Patients with CTO who had viability or ischemia testing before the decision to perform PCI, with post intervention clinical evaluation and/or cardiac testing (LV function, ischemia burden evaluation) |
| I (Intervention) | Successful PCI with documented viability or ischemia testing |
| C (Comparator) | Patients with CTO who did not undergo viability or ischemia testing before PCI, or those where PCI was unsuccessful or not performed |
| O (Outcome) | All-cause mortality, nonfatal myocardial infarction (MI), symptomatic improvement, LV function improvement, and ischemic burden reduction |

**Supplementary Table 3a.** Search strategy - PubMed/MEDLINE

| **Query** | **Search** |
| --- | --- |
| **#1** | (Coronary Occlusion[Title/Abstract]) AND (chronic[Title/Abstract]))) OR ((( "Coronary Occlusion/diagnosis"[Mesh] OR "Coronary Occlusion/diagnostic imaging"[Mesh] OR "Coronary Occlusion/therapy"[Mesh] )) AND "Chronic Disease"[Mesh]) |
| **#2** | ("Heart/diagnostic imaging"[Mesh] OR "Cardiac Imaging Techniques"[Mesh] OR "Ventricular Function, Left"[Mesh] OR "Positron-Emission Tomography"[Mesh] OR "Magnetic Resonance Imaging, Cine"[Mesh] OR "Adenosine"[Mesh] OR "Contrast Media"[Mesh] OR "Dobutamine"[Mesh] OR "Gadolinium"[Mesh] OR "Echocardiography"[Mesh] OR "Fibrosis"[Mesh] OR "Necrosis"[Mesh] OR "Myocardial Ischemia"[Mesh] OR "Myocardial Stunning"[Mesh] OR "Ventricular Dysfunction, Left"[Mesh]) OR (Cardiac Imaging Techniques[Title/Abstract] OR Techniques, Intracardiac Imaging[Title/Abstract] OR Ventricular Function, Left[Title/Abstract] OR Myocardial Perfusion Imaging[Title/Abstract] OR Positron-Emission Tomography[Title/Abstract] OR Positron Emission Tomography Computed Tomography[Title/Abstract] OR Magnetic Resonance Imaging Cine[Title/Abstract] OR Adenosine[Title/Abstract] OR Contrast Media[Title/Abstract] OR Dobutamine[Title/Abstract] OR Gadolinium[Title/Abstract] OR Echocardiography[Title/Abstract] OR Echocardiography, Stress[Title/Abstract] OR Fibrosis[Title/Abstract] OR Necrosis[Title/Abstract] OR Myocardial Ischemia[Title/Abstract] OR Ventricular Dysfunction, Left[Title/Abstract] OR Myocardial Stunning[Title/Abstract] OR Myocardial hibernation[Title/Abstract]) |
| **#3** | (Percutaneous Coronary Intervention[Title/Abstract] OR Angioplasty, Balloon, Coronary[Title/Abstract] OR Atherectomy, Coronary[Title/Abstract] OR Coronary Revascularization, Percutaneous[Title/Abstract] OR Myocardial Revascularization[Title/Abstract] OR "percutaneous coronary intervention"[MeSH Terms] OR "Myocardial Revascularization"[Mesh] ) |
| **#4** | Search #1 AND #2 AND #3 |

**Supplementary Table 3b.** Search strategy - Web of Science Core Collection

| **Query** | **Search** |
| --- | --- |
| **#1** | (Coronary Occlusions AND chronic) OR (Coronary Occlusion AND chronic) (Topic) |
| **#2** | Heart diagnostic imaging OR Cardiac Imaging Techniques OR Ventricular Function Left OR Positron-Emission Tomography OR Magnetic Resonance Imaging Cine OR Adenosine OR Contrast Media OR Dobutamine OR Gadolinium OR Echocardiography OR Echocardiographic OR Fibrosis OR Necrosis OR Myocardial Ischemia OR Myocardial Stunning OR Ventricular Dysfunction Left OR Cardiac Imaging OR Techniques Intracardiac Imaging OR Myocardial Perfusion Imaging OR SPECT OR cMRI OR PET OR Positron-Emission Tomography OR PET CT OR Positron Emission Tomography OR Computed Tomography OR MRI Cine AND Magnetic Resonance Imaging Cine OR Echocardiograph* Stress OR Ventricular Dysfunction* Left OR Myocardial hibernation (Topic) |
| **#3** | percutaneous coronary intervention OR Myocardial Revascularization OR Angioplasty Balloon Coronary OR Atherectomy Coronary OR Coronary Revascularization Percutaneous OR PCI OR Myocardium Revascularization* OR CRP (Topic) |
| **#4** | Search #1 AND #2 AND #3 and Meeting Abstract or Proceeding Paper |

**Supplementary Table 3c.** Search strategy - Embase

| **Query** | **Search** |
| --- | --- |
| **#1** | ('coronary occlusions':ti,ab,kw AND chronic:ti,ab,kw OR ('coronary occlusion':ti,ab,kw AND chronic:ti,ab,kw) OR 'coronary occlusion'/exp |
| **#2** | ('heart diagnostic imaging':ti,ab,kw OR 'cardiac imaging techniques':ti,ab,kw OR 'ventricular function left':ti,ab,kw OR 'magnetic resonance imaging cine':ti,ab,kw OR adenosine:ti,ab,kw OR 'contrast media':ti,ab,kw OR dobutamine:ti,ab,kw OR gadolinium:ti,ab,kw OR echocardiography:ti,ab,kw OR echocardiographic:ti,ab,kw OR fibrosis:ti,ab,kw OR necrosis:ti,ab,kw OR 'myocardial ischemia':ti,ab,kw OR 'myocardial stunning':ti,ab,kw OR 'ventricular dysfunction left':ti,ab,kw OR 'cardiac imaging':ti,ab,kw OR 'techniques intracardiac imaging':ti,ab,kw OR 'myocardial perfusion imaging':ti,ab,kw OR spect:ti,ab,kw OR cmri:ti,ab,kw OR pet:ti,ab,kw OR 'positron-emission tomography':ti,ab,kw OR 'pet ct':ti,ab,kw OR 'positron emission tomography':ti,ab,kw OR 'computed tomography':ti,ab,kw OR 'mri cine':ti,ab,kw) AND 'magnetic resonance imaging cine':ti,ab,kw OR 'echocardiograph* stress':ti,ab,kw OR 'ventricular dysfunction* left':ti,ab,kw OR 'myocardial hibernation':ti,ab,kw) OR ('cine magnetic resonance imaging'/exp OR 'cardiac imaging'/exp OR 'heart left ventricle function'/exp OR 'positron emission tomography'/exp OR 'contrast medium'/exp OR 'adenosine'/exp OR 'dobutamine'/exp OR 'gadolinium'/exp OR 'echocardiography'/exp OR 'heart muscle fibrosis'/exp OR 'necrosis'/exp OR 'heart muscle ischemia'/exp OR 'stunned heart muscle'/exp OR 'myocardial perfusion imaging'/exp OR 'positron emission tomography-computed tomography'/exp OR 'myocardial hibernation'/exp) |
| **#3** | ('percutaneous coronary intervention':ti,ab,kw OR 'myocardial revascularization':ti,ab,kw OR 'angioplasty balloon coronary':ti,ab,kw OR 'atherectomy coronary':ti,ab,kw OR 'coronary revascularization percutaneous':ti,ab,kw OR pci:ti,ab,kw OR 'myocardium revascularization*':ti,ab,kw OR crp:ti,ab,kw) OR ('percutaneous coronary intervention'/exp OR 'heart muscle revascularization'/exp OR 'transluminal coronary angioplasty'/exp OR 'coronary atherectomy'/exp) |
| **#4** | #1 AND #2 AND #3 AND ([article]/lim OR [article in press]/lim OR [preprint]/lim) |

**Supplementary Table 3d.** Search strategy - CENTRAL

| **Query** | **Search** |
| --- | --- |
| **#1** | percutaneous coronary intervention OR Myocardial Revascularization OR Angioplasty Balloon Coronary OR Atherectomy Coronary OR Coronary Revascularization Percutaneous OR PCI OR Myocardium Revascularization* OR CRP in Title Abstract Keyword AND Heart diagnostic imaging OR Cardiac Imaging Techniques OR Ventricular Function Left OR Positron-Emission Tomography OR Magnetic Resonance Imaging Cine OR Adenosine OR Contrast Media OR Dobutamine OR Gadolinium OR Echocardiography OR Echocardiographic OR Fibrosis OR Necrosis OR Myocardial Ischemia OR Myocardial Stunning OR Ventricular Dysfunction Left OR Cardiac Imaging OR Techniques Intracardiac Imaging OR Myocardial Perfusion Imaging OR SPECT OR cMRI OR PET OR Positron-Emission Tomography OR PET CT OR Positron Emission Computed Tomography OR MRI Cine OR Magnetic Resonance Imaging Cine OR Echocardiograph* Stress OR Ventricular Dysfunction* Left OR Myocardial hibernation in Title Abstract Keyword AND (Coronary Occlusions AND chronic) OR (Coronary Occlusion AND chronic) in Title Abstract Keyword - (Word variations have been searched) |

**Supplementary Table 4.** Risk of bias of randomized trials using Cochrane Risk-of-Bias 2 (RoB 2) tool for randomized trials

|  | Randomization process | Deviations from intended interventions | Missing outcome data | Measurement of the outcome | Reported result | Overall risk of bias |
| --- | --- | --- | --- | --- | --- | --- |
| Elias 2017 | Some concerns | Low | Low | Low | Low | Some concerns |
| Mashayekhi 2018 | Low | Low | Low | Low | Low | Low |
| Obendiskiy 2018 | Some concerns | High | High | Low | Low | High |

**Supplementary Table 5.** Risk of bias of non-randomized studies using ROBINS-I tool for non-randomized studies

|  | Confounding factors | Selection of participants | Classification of interventions | Deviations from intended interventions | Missing data | Measurement of outcomes | Reported result | Overall bias |
| --- | --- | --- | --- | --- | --- | --- | --- | --- |
| Baks 2006 | Moderate | Low | Low | Low | Serious | Low | Low | Moderate |
| Bucciarelli-Ducci 2016 | Moderate | Moderate | Low | Low | Moderate | Low | Moderate | Moderate |
| Cardona 2016 | Moderate | Low | Low | Low | Low | Moderate | Low | Low |
| Chen 2019 | Moderate | Low | Low | Low | Low | Low | Low | Low |
| Kiko 2021 | Moderate | Low | Moderate | Low | Moderate | Moderate | Moderate | Moderate |
| Kirschbaum 2008 | Serious | Low | Low | Low | Moderate | Low | Low | Moderate |
| Nakachi 2017 | Moderate | Low | Low | Low | Moderate | Low | Low | Low |
| Paul 2011 | Moderate | Low | Low | Low | Low | Low | Moderate | Low |
| Pujadas 2013 | Moderate | Low | Low | Low | Moderate | Low | Low | Low |
| Rossello 2016 | Moderate | Low | Low | Moderate | Moderate | Low | Low | Moderate |
| Safley 2011 | Moderate | Serious | Low | Low | Serious | Moderate | Low | Serious |
| Schumacher 2019 | Moderate | Low | Low | Low | Moderate | Low | Low | Moderate |
| Schumacher 2020 | Moderate | Low | Low | Low | Low | Low | Moderate | Low |
| Schumacher 2021 | Moderate | Low | Low | Low | Low | Low | Low | Low |
| Schumacher 2021 | Moderate | Low | Low | Low | Moderate | Low | Moderate | Moderate |
| Stuijfzand 2017 | Moderate | Serious | Low | Low | Serious | Moderate | Low | Serious |
| Vitadello 2020 | Moderate | Moderate | Low | Low | Serious | Low | Moderate | Moderate |
| Zhang 2021 | Serious | Serious | Low | Moderate | Serious | Low | Moderate | Serious |

**Supplementary Table 6.** Assessment of Certainty of Evidence Using the GRADE Approach

| **Outcome** | **Number of studies** | **Study Design** | **Risk of Bias** | **Inconsistency** | **Indirectness** | **Imprecision** | **Publication Bias** | **Certainty of Evidence** |
| --- | --- | --- | --- | --- | --- | --- | --- | --- |
| Global Left Ventricular Function | 4 | Observational | Moderate | Low | Low | High | Low | Low |
| Regional Systolic Wall Thickening | 15 | RCTs, Observational | Low to Moderate | Moderate | Moderate | Moderate | Low | Moderate |
| Ischemic Burden | 11 | RCTs, Observational | Low to Moderate | Moderate | Moderate | Low | Low to Moderate | Low to Moderate |
| Symptoms/Quality of life | 5 | RCTs, Observational | Low to Moderate | High | Low | Moderate | Moderate | Moderate |
| Major adverse cardiac events | 4 | RCTs, Observational | Low to Moderate | High | Low | Moderate | Low | Low |


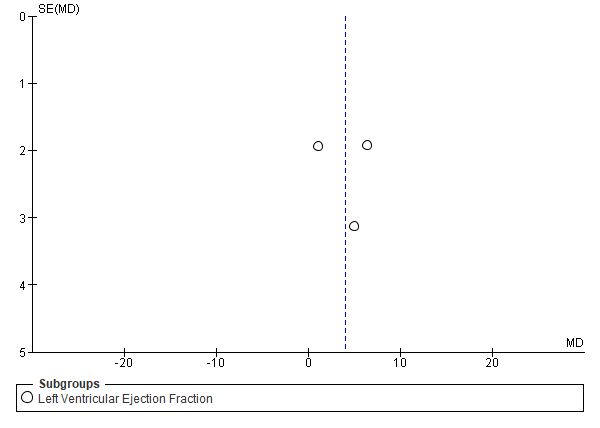


**Supplementary Figure 1.** Left Ventricular Ejection Fraction Funnel Plot


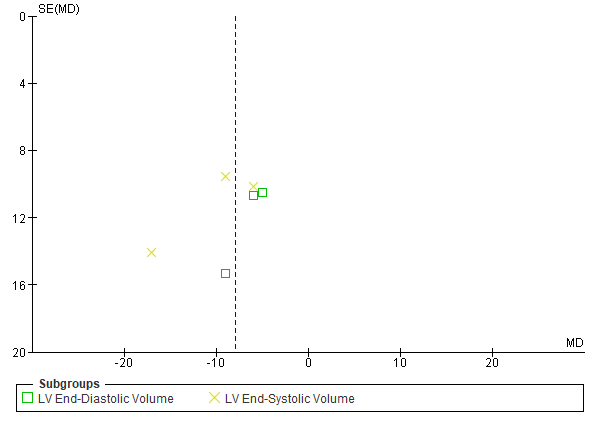


**Supplementary Figure 2.** Left Ventricular (LV) End-Diastolic and End-Systolic Volumes Funnel Plot


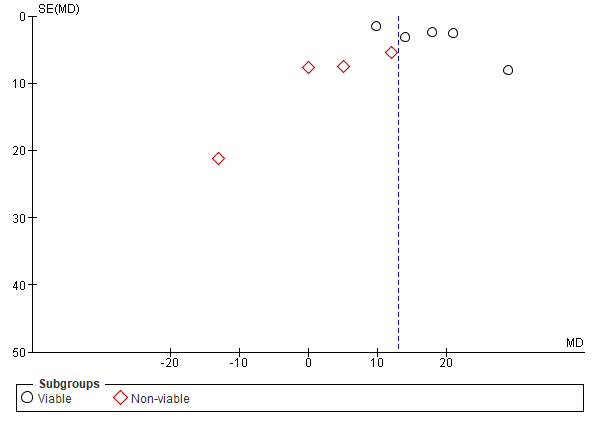


**Supplementary Figure 3.** Regional Segmental Wall Thickness Funnel Plot


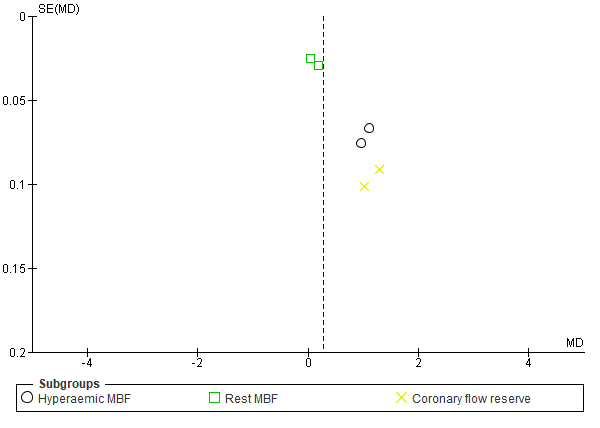


**Supplementary Figure 4.** Hyperaemic and Rest Myocardial Blood Flow (MBF) and Coronary Flow Reserve Funnel Plot


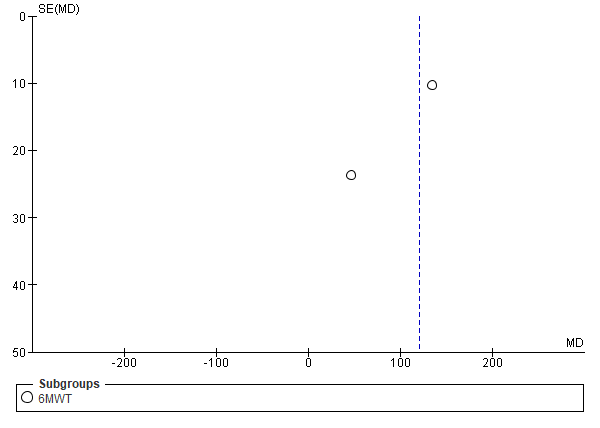


**Supplementary Figure 5.** Six-Minute Walk Test (6MWT) Funnel Plot


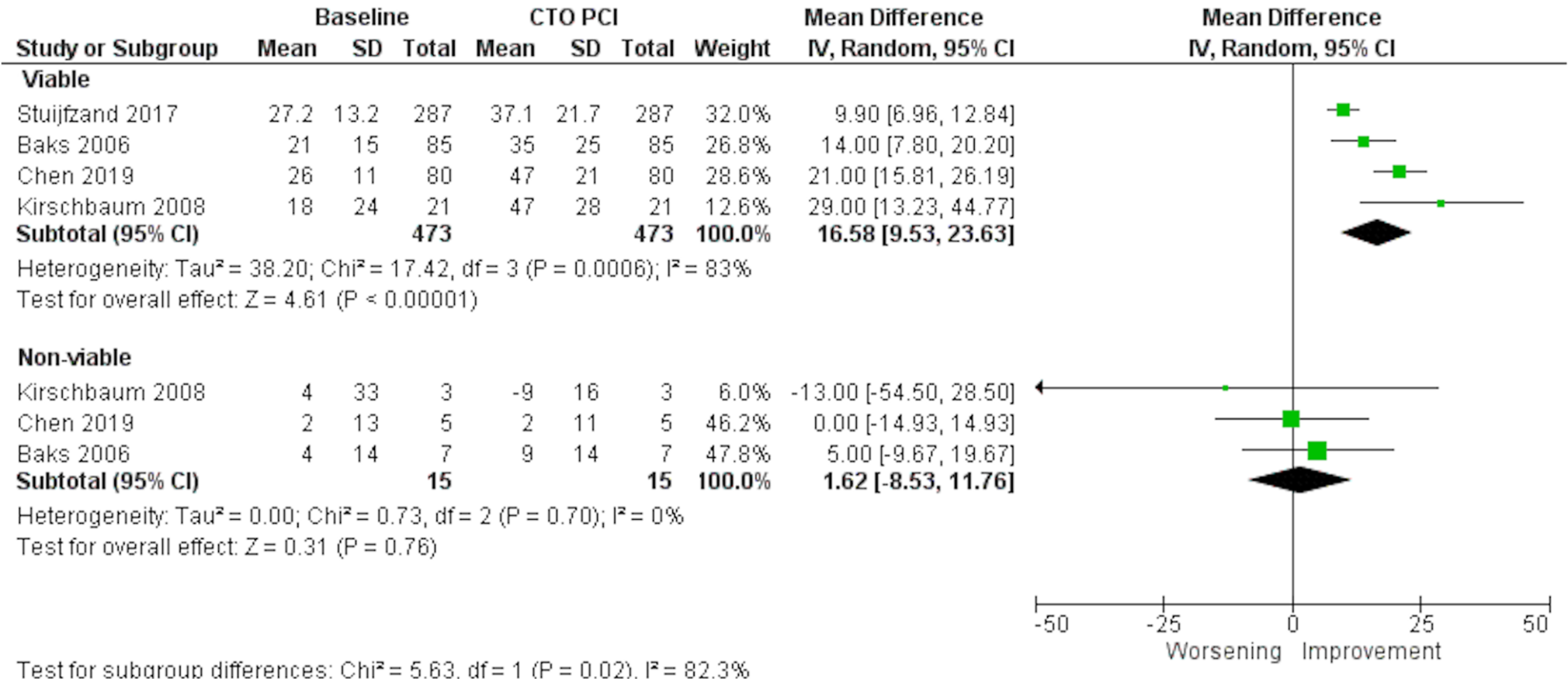


**Supplementary Figure 6.** Sensitivity analysis including only observational studies, evaluating the association of successful chronic total occlusion percutaneous revascularization (CTO-PCI) and segmental wall thickness (SWT) in dysfunctional segments, viable or non-viable

PCI: Percutaneous coronary intervention.


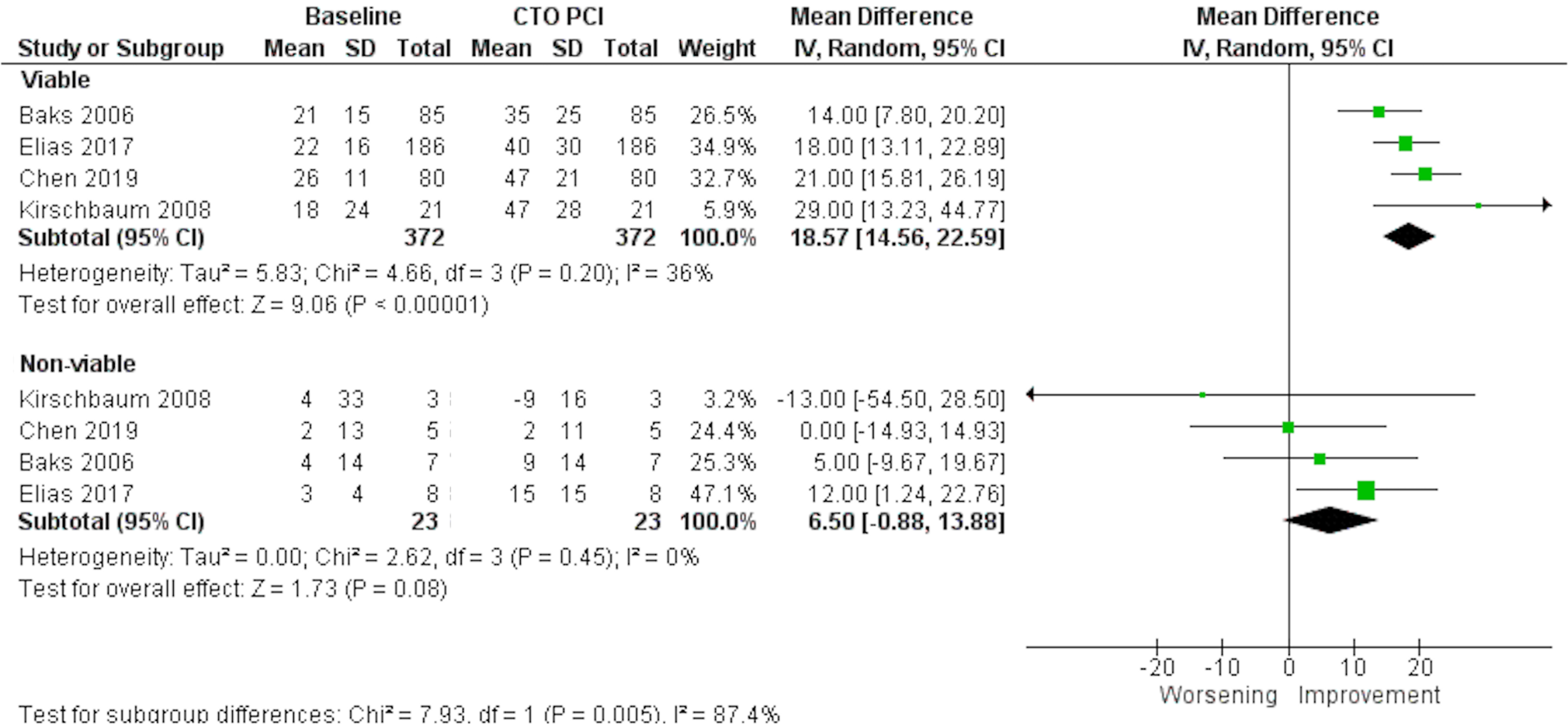


**Supplementary Figure 7.** Sensitivity analysis including only studies that assessed viability/ischemia retrospectively, evaluating the association of successful chronic total occlusion percutaneous revascularization (CTO-PCI) and segmental wall thickness (SWT) in dysfunctional segments, viable or non-viable

PCI: Percutaneous coronary intervention.
